# Morphological and genetic identification of *Halophila* species and a new distribution record of *Halophila nipponica* at the Tanjung Adang Laut shoal, Johor, Malaysia

**DOI:** 10.1371/journal.pone.0309143

**Published:** 2024-10-03

**Authors:** Muhammad Afif Che Alias, Muta Harah Zakaria, Shiamala Devi Ramaiya, Yuzine Esa, Nurul Izza Ab. Ghani, Japar Sidik Bujang

**Affiliations:** 1 Department of Aquaculture, Faculty of Agriculture, Universiti Putra Malaysia, UPM, Serdang, Selangor Darul Ehsan, Malaysia; 2 International Institute of Aquaculture and Aquatic Sciences (I-AQUAS), Universiti Putra Malaysia, Port Dickson, Negeri Sembilan, Malaysia; 3 Department of Crop Science, Faculty of Agricultural and Forestry Sciences, Universiti Putra Malaysia Bintulu Sarawak Campus, Bintulu, Malaysia; 4 Department of Biology, Faculty of Science, Universiti Putra Malaysia, UPM, Serdang, Selangor Darul Ehsan, Malaysia; Karl-Franzens-Universitat Graz, AUSTRIA

## Abstract

The *Halophila* species exhibit complex characteristics due to their high degree of variation across different bioregions. This study delves into the intricate characteristics of *Halophila* species in the Tanjung Adang Laut shoal, Johor, Malaysia, and offers valuable insights through morphological and genetic evidence. Employing internal transcribed sequences (ITS), we investigated the phylogeny of *Halophila* species, revealing distinct clades for *H*. *ovalis*, *H*. *major*, *H*. *spinulosa*, and the newly recorded *H*. *nipponica*. Notably, *H*. *nipponica* from the Tanjung Adang Laut shoal formed a conspecific relationship with its counterparts from Japan and Korea (98.3–98.5% similarity; 5–11 bp differences). Morphologically, distinguishing features, including the ratio of the half-lamina width (1:4.76–6.13 mm) and cross-vein count (4–7 pairs), supported the identification of *H*. *nipponica*. Genetic distance analyses revealed differences between *H*. *nipponica*, *H*. *ovalis*, and *H*. *major*, indicating haplotype diversity. Geographical variations were evident, as *H*. *nipponica* presented unique haplotypes (H24) in its clade. The 47 haplotypes network identified significant mutation sites, providing a comprehensive understanding of genetic and morphological distinctions. In conclusion, this study highlights the intricate characteristics and phylogeny of *Halophila* species in the Tanjung Adang Laut shoal, Johor, and provides valuable insights into their genetic and morphological diversity.

## Introduction

*Halophila* species are submerged marine angiosperms that are widely distributed throughout Tropical Indo-Pacific bioregion [1, 2]. In the genus *Halophila*, 24 species are classified into eight sections on the basis of their morphological and reproductive characteristics [3]. Five sections of distinct *Halophila* species have been reported in Malaysia over the past decade, the Halophila section, *Halophila ovalis* (R. Br.) Hook. *f*., *H*. *major* (Zoll.) Miq. and *H*. *minor* (Zoll.) den Hartog; the Microhalophila section, which is *H*. *beccarii* Aschers.; the Decipientes section, *H*. *decipiens* Ostenfeld; the Spinulosae section, *H*. *spinulosa* (R. Br.) Aschers. and the Tricostatae section, *H*. *tricostata* M. Greenway [4–7]. In a genetic study conducted collaboratively with researchers from Southeast Asia in 2014, a remarkable discovery was made, revealing a new record of seagrass *H*. *major* in diverse locations, including Merambong shoal, Tanjung Adang Laut shoal in Johor, and Manukan Island in Sabah [8]. This revelation expanded the known diversity of *Halophila* species in Malaysia to six. Additionally, a previously identified *Halophila* sp. collected from Teluk Sepinong, Sandakan, Sabah [9], was *H*. *tricostata*, thus increasing the number of recognized *Halophila* species in Malaysia to seven [7, 10].

Ten to twelve species of seagrasses grow in the southern region on calcareous muddy–sandy sub-tidal shoals of Tanjung Adang Laut and Merambong, which have the highest species richness per locality in Peninsular Malaysia or Malaysia, where *Enhalus acoroides* and *H*. *ovalis* have become the dominant species [5, 6, 10]. Thus, the Tanjung Adang Laut shoal has become an interesting location for studying various *Halophila* species. While the majority of research on *Halophila* species in Malaysia has focused on areas such as taxonomy, reproduction, growth dynamics, habitat preferences, and distribution patterns [10–15], there has been a relatively limited focus on genetic studies within this research domain. *H*. *nipponica* is reportedly distributed in warm temperate Korean and Japanese coastal waters and the subtropical northwestern Pacific Ocean [16–19]. This species has morphometric variability in different areas, with the number of cross veins (6-) 7–9 (-10) pairs and the ratio of the distance between the intramarginal vein and lamina margin with 1:1.5–6.5 mm [16]. In Japan, *H*. *nipponica* was observed to have a narrower distribution, whereas in Korea, it has a significantly broader range, often referred to synonymously as *H*. *japonica* [18–20]. Taxonomic identification of *Halophila* species is frequently challenging for researchers because of their high degree of plasticity, leading to potential confusion and misidentification within species and between populations inhabiting similar or different habitats [2, 8, 21]. *Halophila* species exhibit a wide range of leaf shapes and sizes, number of paired cross veins, lengths of petioles, and pigmentation due to environmental factors such as light intensity, sedimentation, and substrates [22–24]. *H*. *major* and *H*. *ovalis* are differentiated by the number of cross veins and the ratio of the distance between the intramarginal vein and lamina margin [8, 25, 26].

Genetic studies play important roles in ecology, conservation, and rehabilitation inference for seagrass ecosystems, fundamentally shaping our understanding and allowing the identification of gene diversity [27]. Instead of the morphology approach, molecular markers have become a determinant for uncertain species. The nuclear ribosomal internal transcribed spacer (ITS1-5.8S–ITS2) region is widely used in seagrass species identification for the Hydrocharitaceae family, including the genera of *Halophila* [28–32], *Enhalus* and *Thalassia* [33, 34]. This marker tends to exhibit high sequence variability and can distinguish closely related species of *Halophila* and be used to study the population-level diversity of species, either within populations [17, 18, 25, 35] or between populations [8, 28]. ITS markers demonstrate high genetic variation and strong genetic structure between *H*. *ovalis* in Southeast Asia and *H*. *ovalis* worldwide [8, 17, 28]. This marker has become a valuable tool for distinguishing between *H*. *ovalis* and *H*. *major* in Malaysia, Vietnam, Indonesia, and Thailand [8, 25, 29]. In the other part of the temperate zone, *H*. *minor*, *H*. *mikii*, *H*. *gaudichaudii*, and *H*. *okinawensis* (originating from the subtropical region) in Japan’s waters are treated as *H*. *ovalis* [17]. *H*. *australis*, initially categorized under the same species name, contradicts the data from ITS sequencing in GenBank, but it was identified as *H*. *major* [28].

The original species classifications of *H*. *ovalis*, *H*. *major*, *H*. *decipiens*, and *H*. *nipponica* were retained on the basis of morphological, reproductive, and molecular genetics data. Only a few genetic studies have been reported in Malaysia on *H*. *ovalis*, *H*. *major*, *H*. *decipiens*, and *H*. *spinulosa* [8, 28, 35]. This underscores the pressing need for comprehensive investigations to fill the knowledge gaps in the genetic diversity of these species. Limitations in terms of genetic divergence and haplotype studies from Malaysia have only been reported for *H*. *ovalis* and *H*. *major* [8, 26], with *H*. *nipponica* and *H*. *spinulosa* remaining unstudied. The determination of *Halophila* species classification relies on the analysis of morphological and molecular genetics data, which constitute the core focus of our studies. Therefore, the present study seeks to unveil the genetic diversity of the *Halophila* genus from the Tanjung Adang Laut shoal and, notably, to describe *H*. *nipponica* as a new distribution record through genetic identification and morphological characteristics from tropical Malaysia. The findings of this study hold significant potential in identifying *Halophila* species, particularly within the Halophila section. Furthermore, comparative interspecies analysis contributes to a better understanding of the divisions within the *Halophila* genus by offering insights into genetic differences, ancestral lineages, and mutations.

## Materials and methods

### Study site, sample collection, and morphology analysis

**Fig 1:**
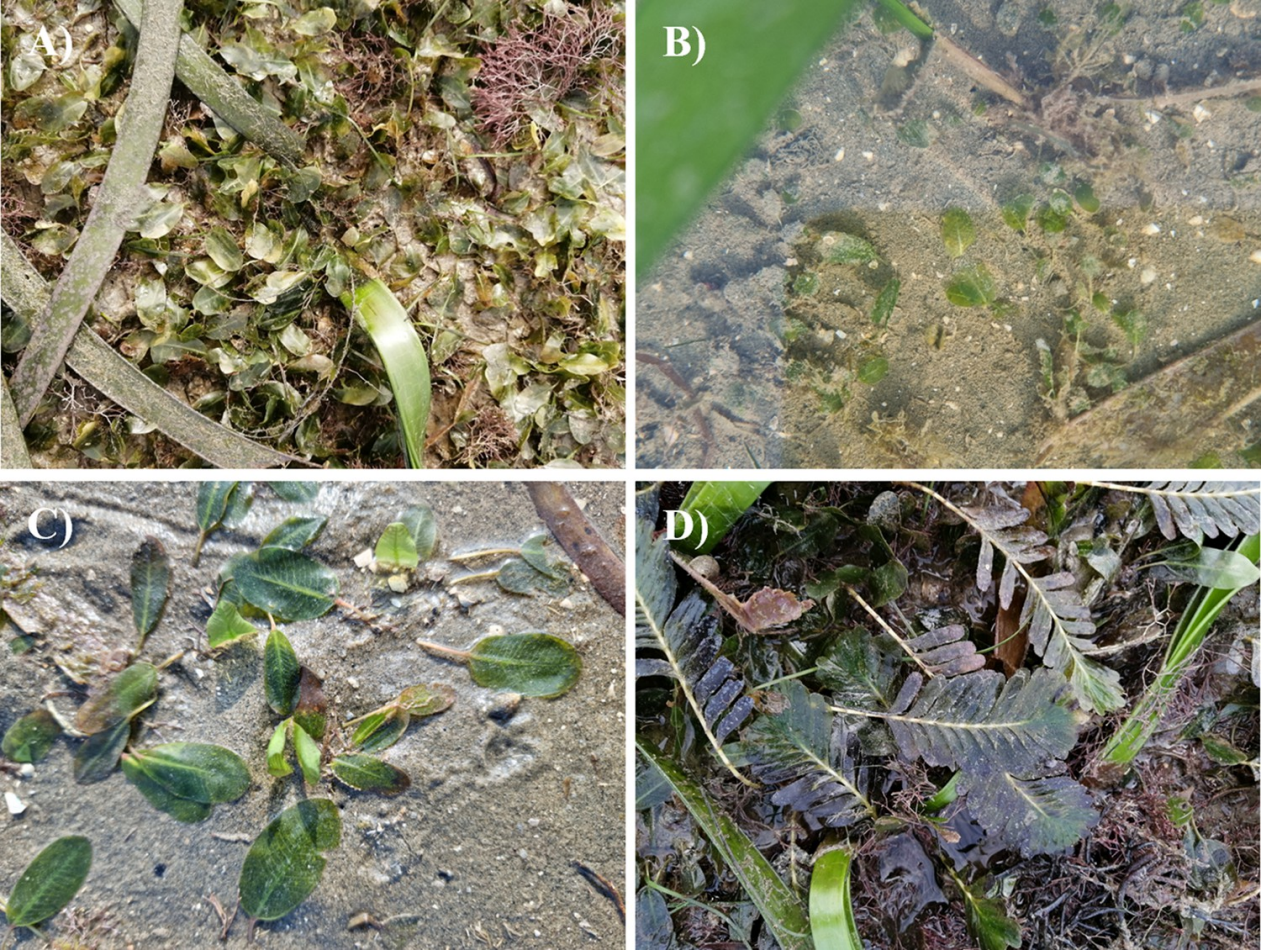
*Halophila* species at Tanjung Adang Laut shoal, Johor. (A) *H*. *ovalis*, (B) *H*. *nipponica*, (C) *H*. *major*, and (D) *H*. *spinulosa*.

*Halophila* plants (Fig 1) were collected from the Tanjung Adang Laut shoal, Johor (1° 19’ 48.0" N 103° 33’ 59.8" E), in March 2022 and June 2022, during low tide on exposed seagrass bed shoal. The salinity and temperature of the seawater were recorded during high tide via the YSI Professional Plus Series Multiparameter. Depending on availability, one to three populations of each species were collected randomly at a distance of 10–30 m to avoid sampling the same clone. Three populations were collected for *H*. *ovalis* and *H*. *major*, two for *H*. *nipponica*, and one for *H*. *spinulosa*. Plants containing leaf blades, petioles, and rhizomes were selected and washed with seawater to remove all the sediment, then placed in sealed polythene plastic bags and brought to the laboratory for further analysis. In the laboratory, samples were divided for morphology analysis and herbarium; young leaves of the plant were preserved with silica gels in a paper bag and placed in a desiccator for DNA extraction. The plants were pressed for herbarium voucher samples [36]. Approximately 13–17 mature leaves were randomly selected for morphometric measurement. Measurements included blade length, blade width, the L:W ratio, the space between the intramarginal vein, the ratio of the distance between the intramarginal vein and lamina margin, the angle of the cross-vein, the distance between the cross veins, and the number of paired cross veins, following key identification by [16, 37]. These measurements were taken via a 5 MP Dino-Lite Edge 3.0 microscope connected to the computer. Statistical analysis of variance (ANOVA, *p* < 0.05) and post-hoc Duncan’s new multiple range test (DNMRT, *p* < 0.05) were performed via IBM SPSS statistic version 27 to compare the vegetative part dimension between populations of *H*. *ovalis*, *H*. *major* and *H*. *nipponica* from the Tanjung Adang Laut shoal.

### DNA extraction, ITS amplification, and sequencing process

The quantity of dried young leaves varied for each species to achieve a homogenized powder weighing 10–20 mg. Approximately 20–30 paired leaves were used for *H*. *ovalis* and *H*. *nipponica*, 5–10 paired leaves were used for *H*. *major*, and 10–15 paired leaflets were used for *H*. *spinulosa*. The plant tissue was homogenized via a mortar [38]. DNA extraction was carried out via the DNeasy® Plant Mini Kit (Qiagen, Inc., Valencia, CA, USA) following the manufacturer’s instructions.

Nine samples of *Halophila* species were collected and subjected to ITS amplification. The region nuclear ribosomal internal transcribed spacer (nrITS), including the 5.8S [28, 39] sequence, was selected for PCR amplification. The primers used were ITS1 (5′-TCCGTAGGTGAACCTGCGG-3′) and ITS4 (5′-TCCTCCGCTTATTGATATGC-3′), which amplified a 700–720 bp sequence. The total volume of 30 μl for PCR components included 10X PCR buffer, 25 mM MgCl_2_, 0.2 mm dNTPs, 5 U/μL Taq DNA Polymerase (Apical Scientific Sdn Bhd, Selangor, Malaysia), 10–30 ng of template DNA, and 1 pmol of each primer. PCR amplification was conducted via a PTC-100® Thermal Cycler (Biosystems, 850 Lincoln Centre Drive, Foster City, California 94404, USA) equipped with a heated lid. The reaction profile involved initial denaturation for 3 min at 95°C, followed by 35 cycles of denaturation for 30 s at 94°C, primer annealing for 30 s at 58°C and extension for 60 s at 72°C, and a final extension for 5 min at 72°C, which was terminated by a final hold at 12°C. The PCR products were observed via 1.2% agarose gel electrophoresis and stained with ethidium bromide. The direct sequencing of the PCR products was carried out via 1st BASE (Selangor, Malaysia) in both directions. PCR replications were conducted for each sample, and the highest-quality sequences were chosen for phylogenetic analysis. The quality of Sanger DNA sequences (Q>20) was determined via FinchTV 1.4 (Geospiza, Inc.; Seattle, WA, USA; http://www.geospiza.com). The consensus sequence was obtained from BioEdit 7.2 (Rutherford House, Manchester Science Parks, Pencroft Way, Manchester M15 6SE, United Kingdom).

### Phylogenetic analyses

Nine sequences from this study (Nos. 1–9) and 28 additional undeposited sequences of *Halophila* species (Nos. 10–37) were obtained from the coauthor Japar Sidik Bujang; these sequences were sourced from various locations and are detailed in Table 1. These sequences were aligned via CLUSTALW in MEGA11 [40] with 63 accessions retrieved from GenBank (http://www.ncbi.nlm.nih.gov/genbank/), as shown in S1 Table. This information is accessible in the public repository of the National Library of Medicine (NLM) at https://www.nlm.nih.gov/accessibility.html. Phylogenetic analyses were conducted via three methods, *i*.*e*., maximum likelihood (ML), neighbour-joining (NJ), and maximum parsimony (MP). The Tamura 3-parameter and gamma-distributed (T92+G) models were chosen on the basis of the lowest Akaike information criterion (AIC) and Bayesian information criterion (BIC) values, indicating the best fit for the 100-sequence dataset [40]. The number of sequence differences was determined via the estimation of the pairwise distance model in MEGA11. The phylogenetics were edited via the iTOL web-based tool (https://itol.embl.de/). The number of haplotypes (N), haplotype diversity (h), and nucleotide diversity (π) were measured within each clade via DnaSP version 6 [41]. Haplotype data were also used to construct a Network 10.2.0.0 (Fluxus Technology) to generate haplotype networks via median-joining [42] for the ITS1-5.8S-ITS2 sequences via their respective alignments. A nonparametric analysis of molecular variance (AMOVA) was performed via ARLEQUIN version 3.5 [43] to assess genetic differentiation among and within clades. The significance of variance components was tested via permutation tests, with 1000 permutations used to generate a null distribution of the test statistics. Fixation indices (F_ST_) were calculated to quantify genetic differentiation, and their significance was also evaluated via permutation tests. A *p*-value of less than 0.05 was considered indicative of significant genetic differentiation.

**Table 1:** Sample collections from this study and additional undeposited sequences from Japar Sidik Bujang (JSB) for phylogenetic analyses.

No.	Species	Location	Sequence label	Source
1.	*H*. *ovalis*	Tanjung Adang Laut shoal, Johor, Malaysia	MY010207TALS1 *H*. *ovalis* (SL)*	This study
2.	*H*. *ovalis*	Tanjung Adang Laut shoal, Johor, Malaysia	MY010207TALS2 *H*. *ovalis* (SL)*	This study
3.	*H*. *ovalis*	Tanjung Adang Laut shoal, Johor, Malaysia	MY010207TALS3 *H*. *ovalis* (SL)*	This study
4.	*H*. *nipponica*	Tanjung Adang Laut shoal, Johor, Malaysia	MY010207TALS1 *H*. nipponica*	This study
5.	*H*. *nipponica*	Tanjung Adang Laut shoal, Johor, Malaysia	MY010207TALS2 *H*. nipponica*	This study
6.	*H*. *major*	Tanjung Adang Laut shoal, Johor, Malaysia	MY010207TALS4 *H*. major*	This study
7.	*H*. *major*	Tanjung Adang Laut shoal, Johor, Malaysia	MY010207TALS5 *H*. major*	This study
8.	*H*. *major*	Tanjung Adang Laut shoal, Johor, Malaysia	MY010207TALS6 *H*. major*	This study
9.	*H*. *spinulosa*	Tanjung Adang Laut shoal, Johor, Malaysia	MY010207TALS5 *H*. spinulosa*	This study
10.	*H*. *ovalis*	Merambong shoal, Johor Malaysia	MY010207MS1 *H*. *ovalis* (RL)#	JSB
11.		Merambong shoal, Johor, Malaysia	MY010207MS2 *H*. *ovalis* (RL)#	JSB
12.		Merambong shoal, Johor, Malaysia	MY010207MS3 *H*. *ovalis* (RL)#	JSB
13.		Merambong shoal, Johor, Malaysia	MY010207MS4 *H*. *ovalis* (SL)#	JSB
14.		Merambong shoal, Johor, Malaysia	MY010207MS5 *H*. *ovalis* (SL)#	JSB
15.		Merambong shoal, Johor, Malaysia	MY010207MS6 *H*. *ovalis* (SL)#	JSB
16.		Merambong shoal, Johor, Malaysia	MY010207MS1 *H*. *ovalis* (BL)#	JSB
17.		Merambong shoal, Johor, Malaysia	MY010207MS2 *H*. *ovalis* (BL)#	JSB
18.		Merambong shoal, Johor, Malaysia	MY010207MS3 *H*. *ovalis* (BL)#	JSB
19.		Tanjung Adang Laut shoal, Johor, Malaysia	MY010207TALS2 *H*. *ovalis* (SL)#	JSB
20.		Tanjung Adang Laut shoal, Johor, Malaysia	MY010207TALS3 *H*. *ovalis* (SL)#	JSB
21.		Tanjung Adang Laut shoal, Johor, Malaysia	MY010207TALS1 *H*. *ovalis* (BL)#	JSB
22.		Tanjung Adang Laut shoal, Johor, Malaysia	MY010207TALS2 *H*. *ovalis* (BL)#	JSB
23.		Tanjung Adang Laut shoal, Johor, Malaysia	MY0107TALS3 *H*. *ovalis* (BL)#	JSB
24.		Seluyong shoal, Johor, Malaysia	MY010207SYS1 *H*. *ovalis* (BL)#	JSB
25.		Seluyong shoal, Johor, Malaysia	MY010207SYS2 *H*. *ovalis* (BL)#	JSB
26.		Seluyong shoal, Johor, Malaysia	MY010207SYS3 *H*. *ovalis* (BL)#	JSB
27.		Mabul Island, Sabah, Malaysia	MY121200PMB *H*. *ovalis#*	JSB
28.		Maiga Island, Sabah, Malaysia	MY121200MG *H*. *ovalis#*	JSB
29.	*H*. *major*	Mabul Island, Sabah, Malaysia	MY121200PMB *H*. *major#*	JSB
30.		Gaya Island, Sabah, Malaysia	MY120100PG *H*. *major#*	JSB
31.		Salakan Island, Sabah, Malaysia	MY121200PS *H*. *major#*	JSB
32.	*H*. *decipiens*	Teluk Pelanduk, Negeri Sembilan, Malaysia	MY050379TP *H*. *decipiens#*	JSB
33.	*H*. *beccarii*	Paka shoal, Terengganu, Malaysia	MY110207SPS *H*. *beccarii#*	JSB
34.	*H*. *spinulosa*	Merambong shoal, Johor, Malaysia	MY010207MS1 *H*. *spinulosa#*	JSB
35.		Merambong shoal, Johor, Malaysia	MY010207MS2 *H*. *spinulosa#*	JSB
36.		Seluyong shoal, Johor, Malaysia	MY010207SYS1 *H*. *spinulosa#*	JSB
37.		Seluyong shoal, Johor, Malaysia	MY010207SYS2 *H*. *spinulosa#*	JSB

RL, red leaves; SL, small leaves; BL, big leaves.

## Results

### Distribution and ecology

**Fig 2:**
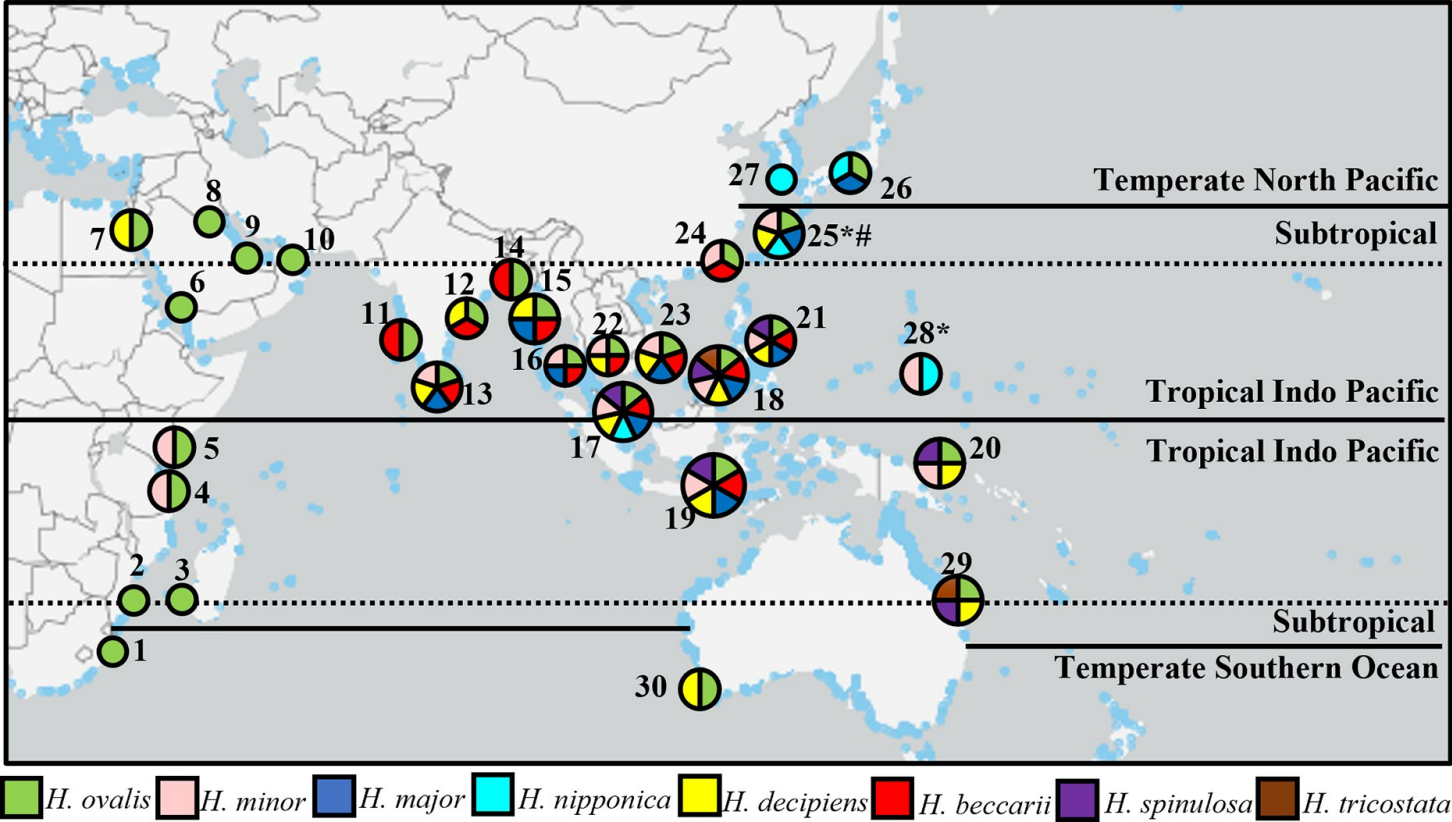
Global distribution of seagrass. Reprinted from [blue]; data from UNEP-WCMC & Short [44] under a CC BY license, with permission from [UNEP-WCMC], original copyright [2021]. Seagrass bioregions adapted from Short et al. [45] include countries with the same *Halophila* species from Malaysia: Temperate North Pacific: 26-Japan, 27-South Korea; Tropical Indo-Pacific: 2-Mozambique, 3-Madagascar, 4-Tanzania, 5-Kenya, 6-South Saudi Arabia, 7-North Saudi Arabia, 8-Kuwait, 9-Bahrain, 10-Yemen, 11-West India, 12-East India, 13-Sri Lanka, 14-Bangladesh, 15-Myanmar, 16-Thailand, 17-West Malaysia, 18-East Malaysia, 19-Indonesia, 20-Papua New Guinea, 21-Philippines, 22-Cambodia, 23-Vietnam, 24-China, 25-South Japan, 28-Guam, 29-East Australia; and Temperate Southern Oceans: 1-South-Africa, 29-West Australia. # *H*. *okinawensis*; * *H*. *gaudichaudii*.

*Halophila nipponica* previously unrecorded in Southeast Asia in the Tropical Indo-Pacific bioregion, was first recorded in Malaysia (Fig 2). This discovery was made in March and June 2022 at the Tanjung Adang Laut shoal, Johor, where small patches were growing in muddy-sandy areas with small leaves *H*. *ovalis* at -2 to -3 m mean sea level (MSL) depth. Salinity and temperature were 28.60–28.92 ppt and 28.70–28.90°C in March; and 27.93–28.85 ppt and 30.20–32.50°C in June, respectively.

### Morphology descriptions

**Fig 3:**
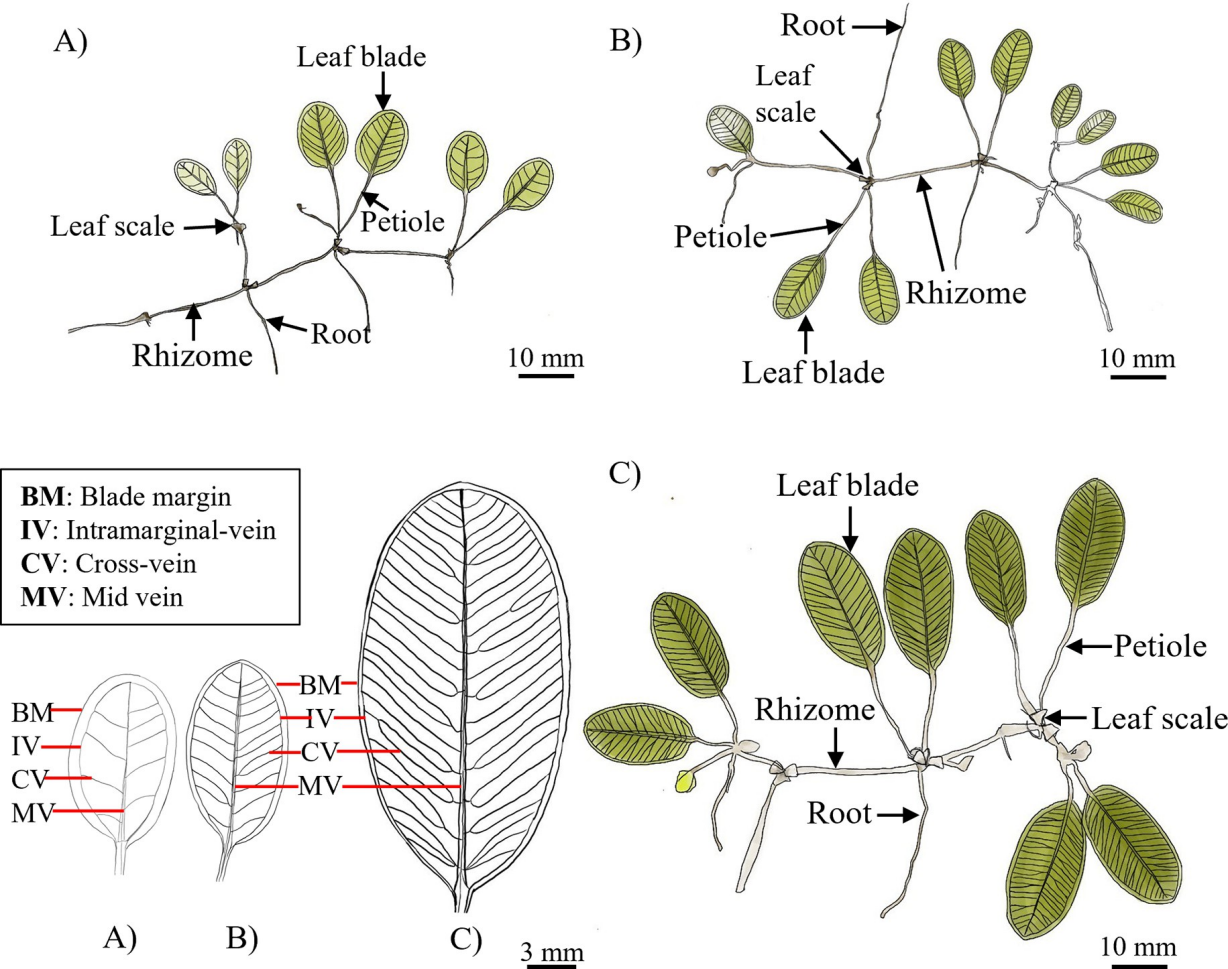
Illustration of *Halophila* species collected from Tanjung Adang Laut shoal, Johor. (A) *H*. *nipponica*, (B) *H*. *ovalis*, and (C) *H*. *major*.

The morphology of *H*. *major*, *H*. *ovalis*, and *H*. *nipponica* collected from the Tanjung Adang Laut shoal is illustrated in Fig 3. The leaf morphologies of *H*. *ovalis*, *H*. *nipponica* and *H*. *major* are shown in Table 2. *H*. *nipponica* had the shortest blade leaves among all populations (*p* < 0.05), ranging from 8.93 ± 0.46 mm to 9.11 ± 0.59 mm, followed by *H*. *ovalis*, ranging from 11.25 ± 1.07 mm to 13.75 ± 1.20 mm (*p* < 0.05). *H*. *major* had the longest blade length, ranging from 30.83 ± 2.58 mm to 38.36 ± 2.22 mm. The blade width in *H*. *nipponica* population 2 was narrower, while that in *H*. *nipponica* population 1 was similar to that in *H*. *ovalis* population 2, followed by that in *H*. *ovalis* population 3. However, the blade width of *H*. *major* was significantly wider than that of both *H*. *ovalis* and *H*. *nipponica* (*p* < 0.05). *H*. *nipponica* was differentiated by having the lowest number of paired cross-veins, ranging from 4 to 7 pairs, followed by *H*. *ovalis*, with 7 to 14 pairs, and *H*. *major*, with 13 to 18 pairs (*p* < 0.05). This differentiation was further supported by the ratio of the space between the intramarginal vein and the blade margin (r:R), which was 1:4.76–6.13 mm for *H*. *nipponica*, 1:8.00–14.16 mm for *H*. *ovalis*, and 1:17.33–36.97 mm for *H*. *major*. The distance between the cross-veins of *H*. *nipponica* and *H*. *ovalis* showed no significant difference, ranging from 0.99 ± 0.52 mm to 1.55 ± 0.62 mm, while *H*. *major* had a slightly greater distance, ranging from 2.53 ± 1.38 mm to 3.25 ± 1.92 mm.

**Table 2:** Vegetative comparison of *Halophila* species at Tanjung Adang Laut shoal, Johor.

Species/(no. of sample)	Vegetative structure dimension								
	BL (mm)	BW (mm)	ratio BL: BW (mm)	R (mm)	r (mm)	ratio r:R (mm)	NCV	ACV	DBCV (mm)
*H*. *nipponica* Population 1 (n = 15)	9.11±0.59^a^ (8.21–10.35)	6.33±0.27^ab^ (5.95–7.00)	1.44±0.06^a^ (1.34–1.58:1)	3.29±0.18^ab^ (3.06–3.57)	0.61±0.05^d^ (0.52–0.69)	5.46±0.41^a^ (1:4.76–6.13)	5.67±1.18^a^ (4–7)	66.20°±8.65^c^ (50.25°–82.86°)	1.55±0.62^a^ (0.59–2.50)
*H*. *nipponica* Population 2 (n = 15)	8.93±0.46^a^ (8.32–9.92)	6.07±0.56^a^ (5.18–7.26)	1.48±0.11^a^ (1.29–1.64:1)	3.17±0.31^a^ (2.61–3.75)	0.59±0.06^d^ (0.52–0.70)	5.36±0.39^a^ (1:4.89–6.02)	5.60±0.91^a^ (4–7)	65.29°±6.48^c^ (52.37°–73.51°)	1.41±0.66^a^ (0.43–2.29)
*H*. *ovalis* Population 1 (n = 13)	11.25±1.07^b^ (9.86–13.13)	6.43±0.52^ab^ (5.70–7.67)	1.75±0.11^b^ (1.57–1.90:1)	3.28±0.28^ab^ (2.88–3.91)	0.33±0.04^a^ (0.25–0.42)	10.12±1.35^b^ (1:8.56–13.56)	10.62±1.85^c^ (7–14)	65.74±7.64^c^ (50.55°–77.13°)	0.99±0.52^a^ (0.13–1.81)
*H*. *ovalis* Population 2 (n = 15)	11.94±1.25^b^ (9.55–14.38)	7.08±0.69^b^ (5.31–8.20)	1.69±0.13^b^ (1.45–1.87:1)	3.65±0.35^b^ (2.84–4.24)	0.37±0.04^b^ (0.32–0.46)	9.85±1.12^b^ (1:8.29–11.46)	9.47±1.25^b^ (8–11)	66.51±7.93^c^ (52.39°–86.89°)	1.08±0.58^a^ (0.24–1.98)
*H*. *ovalis* Population 3 (n = 15)	13.75±1.20^c^ (11.77–15.32)	8.05±0.97^c^ (6.62–10.15)	1.72±0.19^b^ (1.39–1.96:1)	4.12±0.56^c^ (3.12–5.42)	0.37±0.06^b^ (0.28–0.49)	11.28±1.86^b^ (1:8.00–14.16)	10.47±1.25^c^ (8–12)	63.00±5.92^bc^ (53.04°–70.9°)	1.23±0.57^a^ (0.31–1.98)
*H*. *major* Population 1 (n = 15)	30.83±2.58^d^ (26.81–37.95)	17.67±1.49^d^ (15.10–21.65)	1.75±0.09^b^ (1.62–1.93:1)	9.14±0.75^d^ (7.99–11.30)	0.45±0.05^c^ (0.40–0.59)	20.53±1.99^c^ (1:17.98–24.08)	14.73±0.88^d^ (13–16)	56.86±4.15^a^ (50.22°–64.79°)	2.53±1.38^b^ (0.51–4.38)
*H*. *major* Population 2 (n = 13)	38.36±2.22^f^ (35.11–42.30)	19.40±1.86^e^ (15.80–22.03)	2.00±0.23^c^ (1.69–2.36:1)	10.08±0.82^e^ (8.59–11.22)	0.47±0.09^c^ (0.30–0.58)	22.30±5.54^c^ (1:17.33–36.80)	14.69±1.37^d^ (13–18)	58.51±5.37^a^ (48.95°–66.37°)	3.25±1.92^b^ (0.77–5.73)
*H*. *major* Population 3 (n = 17)	33.91±2.50^e^ (28.82–37.25)	19.14±1.46^e^ (15.21–20.86)	1.78±0.13^b^ (1.50–2.03:1)	9.98±0.72^e^ (8.31–11.09)	0.40±0.06^b^ (0.30–0.55)	25.59±4.39^d^ (1:18.75–36.97)	15.53±1.18^d^ (14–18)	65.04±5.96^c^(53.59°–72.05°)	2.72±1.50^b^ (0.46–4.66)

BL, Blade length; BW, Blade width; R, half lamina width; r, distance between intramarginal and blade margin; NCV, number of paired cross-veins; ACV, angle of the cross vein; DBCV, distance between cross veins. The varying superscript alphabet in the same column demonstrates the contrast at *p* < 0.05 (ANOVA Duncan new multiple range test). Value is given as mean ± standard deviation and range in parentheses.

**Fig 4:**
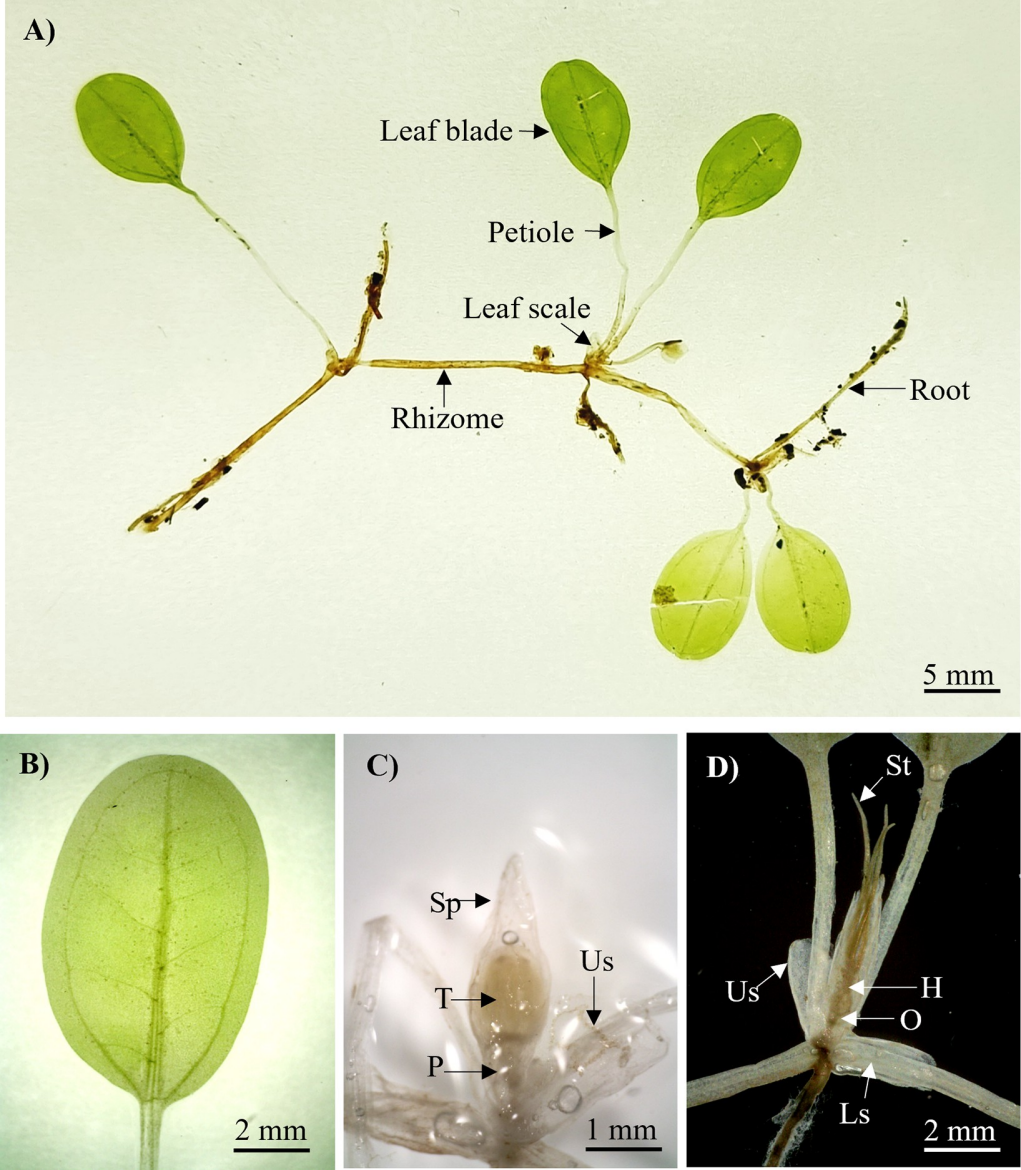
Morphology features of *H*. *nipponica* from Tanjung Adang Laut shoal, Johor. (A) The habit of *H*. *nipponica*, (B) Leaf blade, (C) Male flower, and (D) Female flower. Us, upper scale; Ls, lower scale; Sp, spathe; T, tepal; P, pedicel; St, style; H, hypanthium; O, ovary.

*Halophila nipponica* (Fig 4) was identified as a dioecious plant with a fragile narrow rhizome, 0.40–0.60 mm thick and rhizome internodes 10.00–20.00 mm long. Each node had one unbranched root with numerous root hairs. The leaf scales (Fig 4A) were transparent, folded, and convex, with entire margins of approximately 2.35–2.75 × 2.86–3.60 mm. The petioles were 10.00–12.50 mm long and 0.40–0.45 mm thick. The leaves were light green, elliptical, and orbicular in shape (Fig 4B), with entire margins of 8.21–10.35 mm in length and 5.18–7.26 mm in width, an L:W ratio of 1.29–1.64:1 mm and a half lamina width of 2.61–3.75 mm (Table 2). The apex of the leaf was rounded, whereas the leaf base was rounded or oblique. The space between the intramarginal vein and the blade margin was 0.52–0.70 mm. The ratio of the distance between the intramarginal vein and the lamina margin was 1:4.76–6.13 mm. The number of paired cross-veins ranged from 4 to 7, with a distance between cross-veins of 0.43–2.50 mm and an angle of 50.25°–82.86°. The young mature male flower (Fig 4D) consisted of a transparent spathe, that was 1.27–2.18 mm long and 0.99–1.14 mm wide, three whitish tepals that were 1 mm long and 0.52 mm wide, and a 1 mm long pedicel. The female flower (Fig 4E) had an ovary of 0.87–1.20 × 0.67–1.00 mm, a hypanthium 0.36–5.08 mm long, and three styles of 4.60–7.20 mm long.

**Fig 5:**
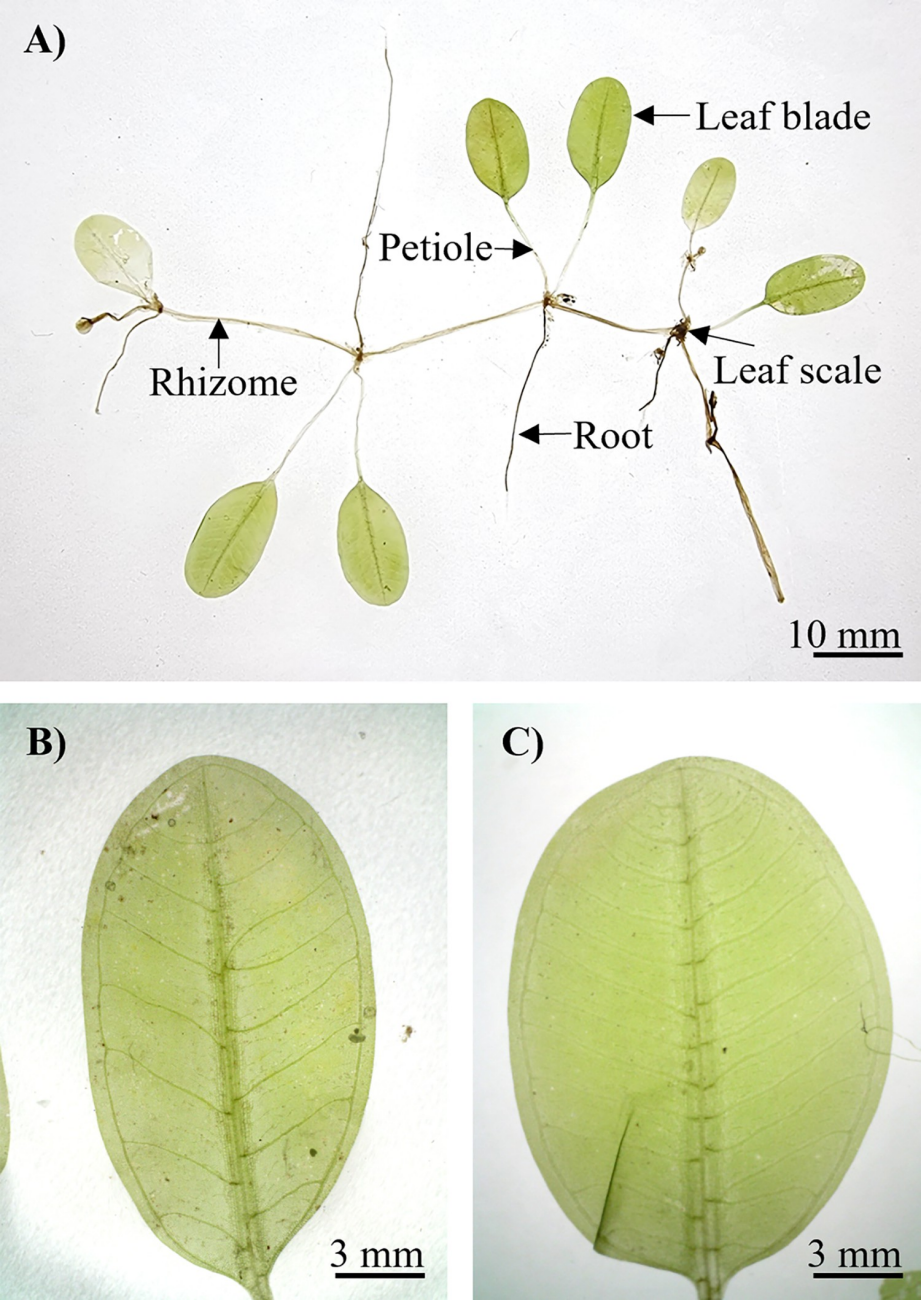
Morphology features of *H*. *ovalis* from Tanjung Adang Laut shoal, Johor. (A) The habit of *H*. *ovalis*, (B) Elliptic leaf, and (C) Ovate leaf.

*Halophila ovalis* (Fig 5) is a dioecious plant with a creeping, fragile, narrow rhizome that is 0.50–1.40 mm thick and rhizome internodes 17.00–25.00 mm long. Each node had a single unbranched root with numerous root hairs. The leaf scales were transparent, folded, and convex, with entire margins of approximately 3.20–3.29 × 2.62–3.64 mm. The petioles were 8.00–17.00 mm long and 0.40–0.45 mm thick. The leaf blades were light green, elliptical (Fig 5B), and ovate (Fig 5C) in shape, with entire margins of 9.55–15.32 mm in length and 5.31–10.15 mm in width, with an L:W ratio of 1.39–1.96:1 mm and a half lamina width of 2.84–5.42 mm (Table 2). The leaf apex was obtuse and rounded, whereas the base was rounded or oblique. The space between the intramarginal vein and the blade margin was 0.25–0.49 mm, with a distance between the intramarginal vein and lamina margin of 1:8.00–14.16 mm. The number of paired cross-veins ranged from 7 to 14, with single- and double-branch veins. The distance between cross veins was 0.13–1.98 mm, at an angle of 50.55°–86.89°. Male and female flowers, as well as fruits, were absent from the collected samples.

**Fig 6:**
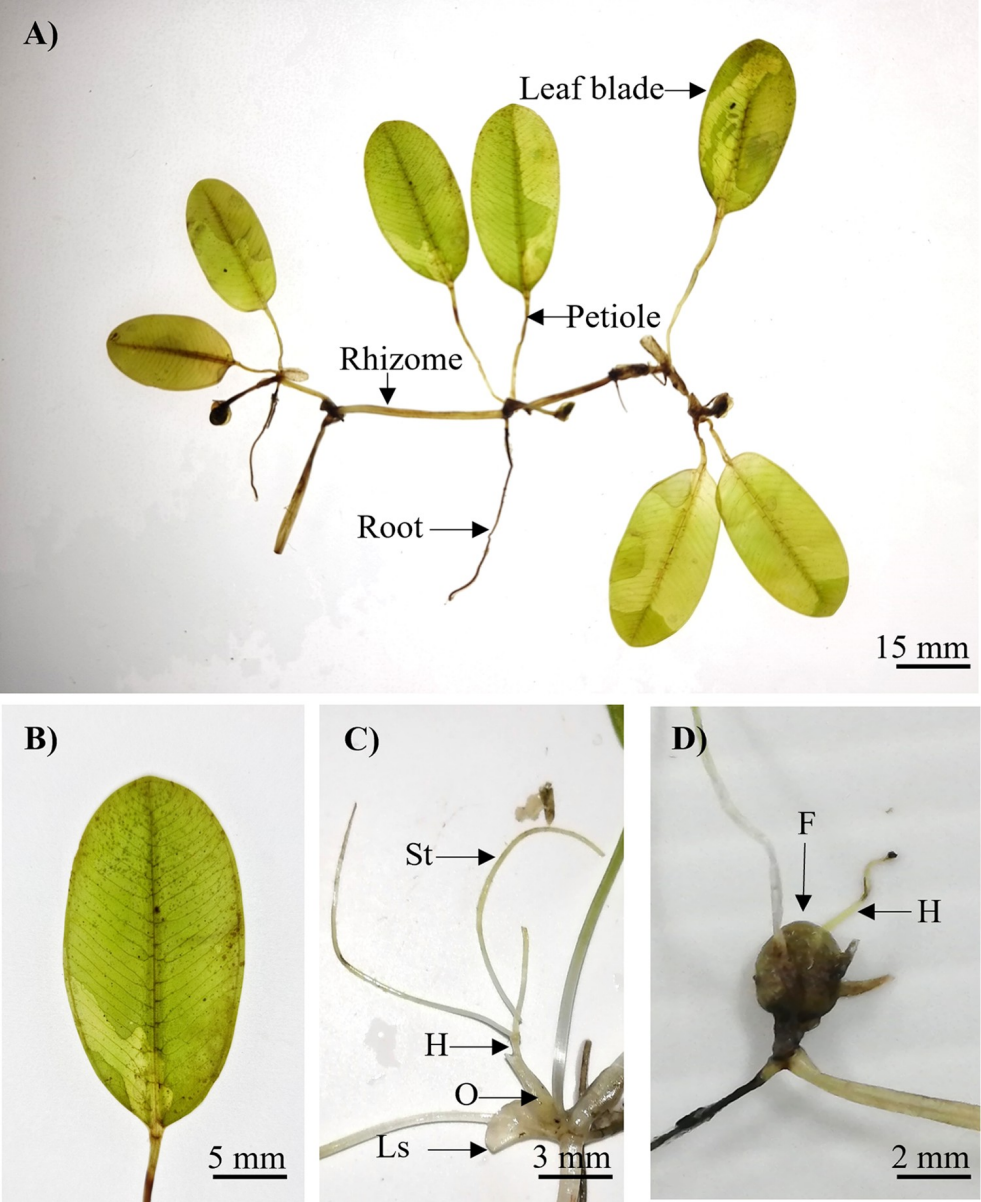
Morphology features of *H*. *major* from Tanjung Adang Laut shoal, Johor. (A) The habit of *H*. *major*, (B) Leaf blade, (C) Female flower, and (D) Fruits. Ls, lower scale; St, style; H, hypanthium; O, ovary; F, fruit.

*Halophila major* (Fig 6) is a dioecious plant with a thick whitish rhizome that is 1.00–1.80 mm in diameter and rhizome internodes that are 11.30–23.00 mm long. Each node had one unbranched root with numerous root hairs. The leaf scales were transparent, folded, and convex, with 3.00–4.00 × 2.00–2.50 mm. The petioles were 30.00–38.00 mm long and 0.80–1.00 mm thick. The leaf blades (Fig 6B) were dark green, elliptical, ovate, and oblong with entire margins of 26.81–42.30 mm in length and 15.10–22.03 mm in width, with an L:W ratio of 1.50–2.36:1 mm and a half lamina width of 7.99–11.30 mm (Table 2). The leaf apex was obtuse and rounded, whereas the base was rounded or oblique. The space between the intramarginal vein and blade margin was 0.30–0.59 mm, with a ratio of the distance between the intramarginal vein and lamina margin of 1:17.33–36.97 mm. The number of paired cross-veins ranged from 13 to 18, with 2 to 5 branched veins. The distance between cross veins was 0.46–5.73 mm at an angle of 48.22°–72.05°. The male flower was absent. The female flower (Fig 6C) had an ovary of 2.00–2.15 × 1.00–1.20 mm, a hypanthium 4.00–7.00 mm long, and styles 14.00–20.00 mm long. The fruit (Fig 6D) was 2 mm in diameter.

**Fig 7:**
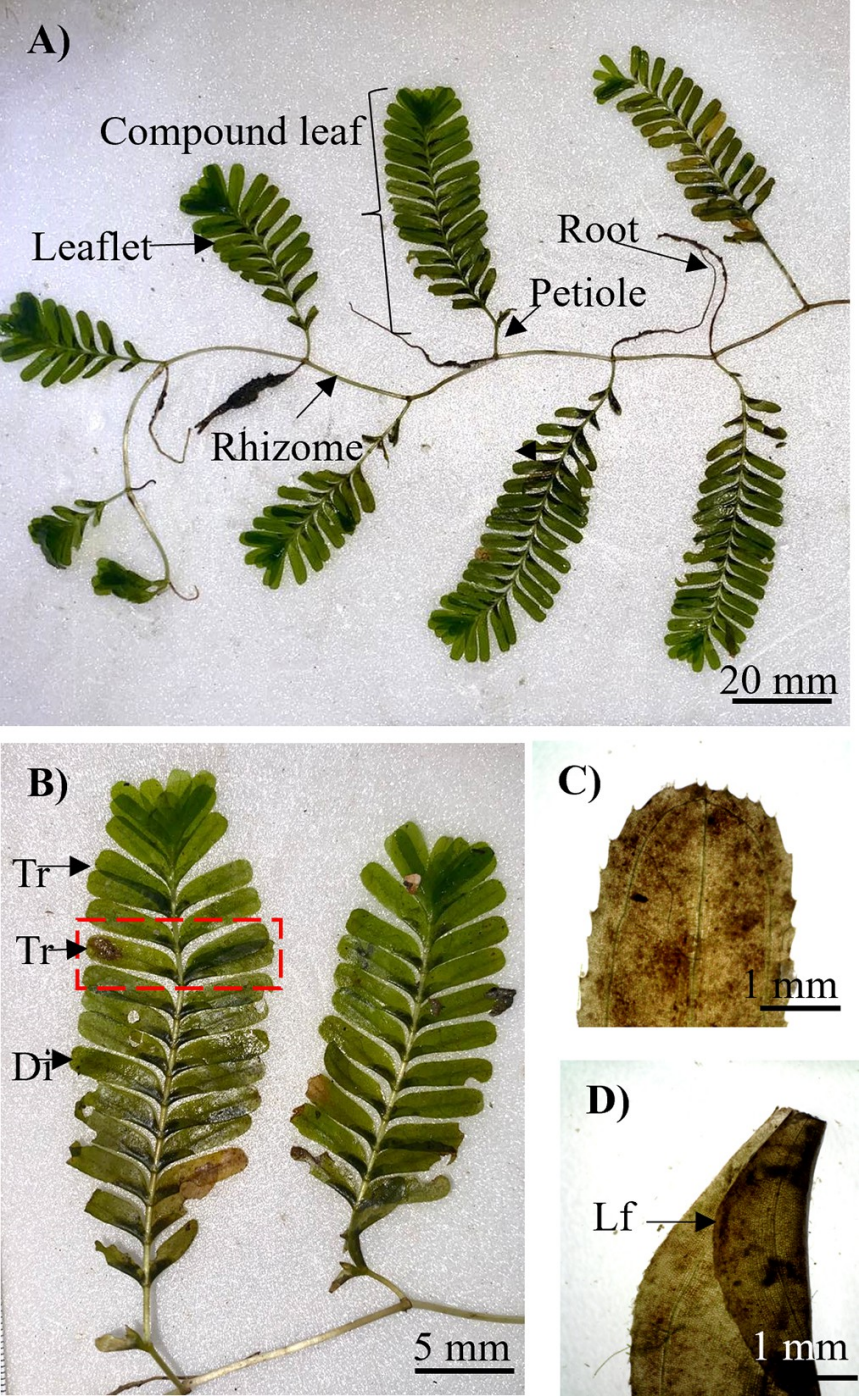
Morphology features of *H*. *spinulosa* from Tanjung Adang Laut shoal, Johor. (A) Vegetation habit of *H*. *spinulosa*, (B) Compound leaf, (C) Serrate leaflet, and (D) Lower leaflet with leaf folded. Tr, tristichous; Di, distichous.

*Halophila spinulosa* (Fig 7) is a dioecious plant with woody rhizomes that are 0.85–1.90 mm thick and rhizome internodes that are 15.70–35.10 mm long. Each rhizome node had an unbranched root with numerous root hairs and a nonflowering or flowering erect shoot bearing compound leaves (Fig 7A) with leaflets. The leaflets were arranged distichously and tristichously (Fig 7B). The leaflet blades were dark green, oblong-linear, 7.00–16.00 mm long and 2.30–4.00 mm wide, with 4–7 pairs of cross-veins. The leaflet margin was folded near the base one side (Fig 7D and 7E). The leaf tip was rounded and serrated. The midrib was conspicuous and united at the top with intramarginal veins. Male and female flowers, as well as fruit, were absent from the collected sample.

### Phylogenetic analysis

**Fig 8:**
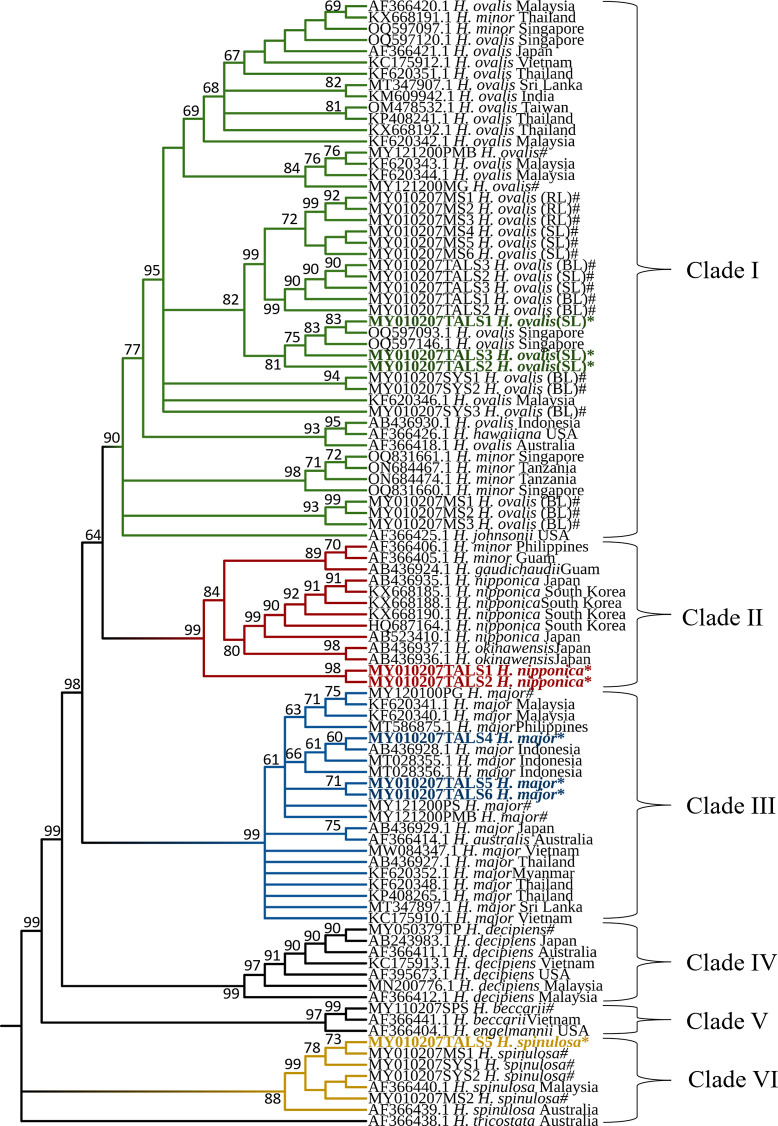
The phylogenetic tree of *Halophila* species is inferred from maximum parsimony analysis. 1000 replication bootstrap using 651 base pairs of nrDNA, including ITS1, 5.8S rDNA, and ITS2. Bootstrap support values above 50% are shown on branches. Over 70% of the bootstrap value is threshold confidence. (*), sample of this study; (#), undeposited sequences from Japar Sidik Bujang.

Phylogenetic analyses were conducted via three methods, *i*.*e*., maximum-likelihood (ML), neighbor-joining (NJ), and maximum parsimony (MP), employing Tamura 3-parameter and gamma-distributed (T92+G) models with a dataset of 100 sequences. The MP method yielded the most suitable sequence alignment, resulting in six well-supported clades (Clades I-VI) among the *Halophila* species, as presented in Fig 8.

Clade I included *H*. *ovalis*, which clustered with a 90% bootstrap value alongside *H*. *minor*, *H*. *hawaiiana*, and *H*. *johnsonii*. Within Clade I, the small-leaved *H*. *ovalis* from this study clustered with all *H*. *ovalis* samples from the Tanjung Adang Laut shoal, and *H*. *ovalis* (SL) and *H*. *ovalis* (RL) from the Merambong shoal undeposited sequences from Japar Sidik Bujang. This is also supported by an 82% bootstrap value and a subgroup of Singapore samples with an 81% bootstrap value. *H*. *ovalis* (MY121200PMB# and MY121200MG#) from Sabah were clustered with *H*. *ovalis* from Malaysia (KF620343 and KF620344). In Clade II, *H*. *nipponica* from this study clustered with a strong 99% supported bootstrap value and was separated from the subgroup comprising *H*. *nipponica*, *H*. *okinawensis*, *H*. *gaudichaudii*, and *H*. *minor*.

*H*. *major* in this study were clustered in Clade III with 99% bootstrap supported value escorted samples from Malaysia, Indonesia, Thailand, Vietnam, Myanmar, Sri Lanka, Japan, and *H*. *australis* from Australia. *H*. *major* of this study was subgrouped with Indonesia and separated with *H*. *major* from Sabah, Malaysia with a 61% bootstrap value. Clade IV clustered *H*. *decipiens* from Malaysia, Vietnam, Japan, Australia, and the USA according to a 99% bootstrap value. Clade V received 97% bootstrap values for *H*. *beccarii* from Malaysia and Vietnam and *H*. *engelmannii* from the USA. In Clade VI, *H*. *spinulosa* from Malaysia was clustered with Australia by 88%, supporting the bootstrap value.

### Sequence characteristics and genetic divergence

The final alignment of nine sequences from this study, represented in Table 3, consisted of 651 position datasets (including gaps), resulting in a total number of nucleotides ranging from 609 to 621 position datasets. The percentage of guanine-cytosine content (%G~C content) of *H*. *ovalis* at the Tanjung Adang Laut shoal, Johor, was identical among the three collection points (63.28%), whereas respectively, while *H*. *nipponica* presented a lower percentage of G~C content (62.91%). *H*. *major* contained 64.9% and 65.06% G~C content, whereas *H*. *spinulosa* presented the lowest content, at only 58.30%.

**Table 3:** Nucleotide composition (%).

Samples of study	Nucleotide Composition (%)				Total number of nucleotides
	T	C	A	G	
MY010207TALS1 *H*. *ovalis*	19.65	32.85	17.07	30.43	621
MY010207TALS2 *H*. *ovalis*	19.65	32.85	17.07	30.43	621
MY010207TALS3 *H*. *ovalis*	19.65	32.85	17.07	30.43	621
MY010207TALS1 *H*. *nipponica*	19.84	31.94	17.26	30.97	620
MY010207TALS2 *H*. *nipponica*	19.84	31.94	17.26	30.97	620
MY010207TALS4 *H*. *major*	18.52	33.98	16.43	31.08	621
MY010207TALS5 *H*. *major*	18.52	33.98	16.59	30.92	621
MY010207TALS6 *H*. *major*	18.52	33.98	16.43	31.08	621
MY010207TALS5 *H*. *spinulosa*	21.67	29.89	20.03	28.41	609

T, thymine; C, cytosine; A, adenine; G, guanine. Analysed using MEGA11.

The analysis of sequence divergences involved 100 nucleotide sequences, as shown in S2 Table, via the Tamura-3 parameter model over 1000 replications to locate pairwise distances. In the present study, *H*. *nipponica* and *H*. *ovalis* presented a significant difference of 0.040 in sequence distance. The ITS sequences of *H*. *ovalis* were not different in terms of genetic distance between their populations and were identical to those of *H*. *ovalis* from Singapore (OQ597146 and OQ597093) (Table A in S3 Table). A comparison with other *H*. *ovalis* sequences revealed genetic differences between 0.002 and 0.027 (1–17 bp differences), with the highest divergences between *H*. *ovalis* (red leaf) from the Merambong shoal. The observed sequence divergence of *H*. *nipponica* was 0.015 (10 bp difference) compared with that of its temperate Japanese counterparts and 0.015–0.017 (10–11 bp difference) compared with that of its South Korean counterparts (Table B in S3 Table).

A comparison of the pairwise distance of the *H*. *major* ITS from the Tanjung Adang Laut shoal, Johor, revealed minimal sequence differences of 0.002–0.003 (1–2 bp differences) between populations (Table C in S3 Table), in comparison with other *H*. *major* sequences, which presented variations of 0.002–0.011 (1–7 bp differences). *H*. *spinulosa* at the Tanjung Adang Laut shoal demonstrated low sequence divergence towards species from Malaysia (0.000–0.002; 0–1 bp differences) but notably greater differences than *H*. *spinulosa* from Australia (0.017; 11 bp differences) and *H*. *tricostata* (0.031; 20 bp differences), as shown in Table D in S3 Table. Interspecies pairwise distance comparisons between Clades I (*H*. *ovalis*) and II (*H*. *nipponica*) revealed an average sequence divergence of 0.032 (96.8% similarity; 21 bp differences), as shown in Table 4. The interspecies pairwise distance between Clades III (*H*. *major*) and I was lower than that between Clades II, with values of 0.044 (95.6% similarity; 29 bp difference) and 0.051 (94.9% similarity; 33 bp difference), respectively. This trend continued with Clades IV, V, and VI. Clade VI, containing *H*. *spinulosa*, showed considerable sequence distance from all other clades, with values ranging from 0.144 to 0.165 (83.5–85.6% similarity; 94–107 bp differences).

**Table 4:** Genetic divergence between clades (interspecies pairwise distance).

	Clade_I	Clade_II	Clade_III	Clade_IV	Clade_V	Clade_VI
**Clade_I**		0.007	0.009	0.012	0.018	0.021
**Clade_II**	0.032		0.010	0.013	0.018	0.022
**Clade_III**	0.044	0.051		0.014	0.017	0.022
**Clade_IV**	0.070	0.076	0.082		0.018	0.020
**Clade_V**	0.123	0.126	0.117	0.123		0.020
**Clade_VI**	0.155	0.165	0.165	0.150	0.144	

Standard error estimate(s) were shown above the diagonal.

### Haplotype diversity of *Halophila* species

Further analyses were conducted to increase the amount of supporting data on the determination of haplotype diversity and its differences between other *Halophila* species. A total of 100 sequences were estimated from six different clades (Table 5). The total nucleotide diversity (π) for all clades was 0.04950. The total haplotype diversity (Hd) was 0.968 from the 47 haplotype numbers (H). Clade V presented the highest haplotype diversity (1.000), where *H*. *engelmannii* was grouped with *H*. *beccarii*, followed by Clades III, I, II, and IV (0.914, 0.908, 0.859, and 0.524, respectively), and the lowest was in Clade VI (0.464). The number of segregating sites (S) of Clade IV was the lowest, with six sequence site differences, while it was the highest for Clade V (55 sites).

**Table 5:** Summarize of *Halophila* species by clade.

Clade	N	H	Hd	π	S
I	48	16	0.908	0.01338	31
II	13	8	0.859	0.01072	16
III	21	14	0.914	0.00574	20
IV	7	3	0.524	0.00327	6
V	3	3	1.000	0.06997	55
VI	8	3	0.464	0.00941	19
Total Data Estimates	100	47	0.968	0.04950	159

N, sample size; H, number of haplotypes observed; Hd, haplotype diversity; π, estimate nucleotide diversity; S, number of segregating sites.

**Fig 9:**
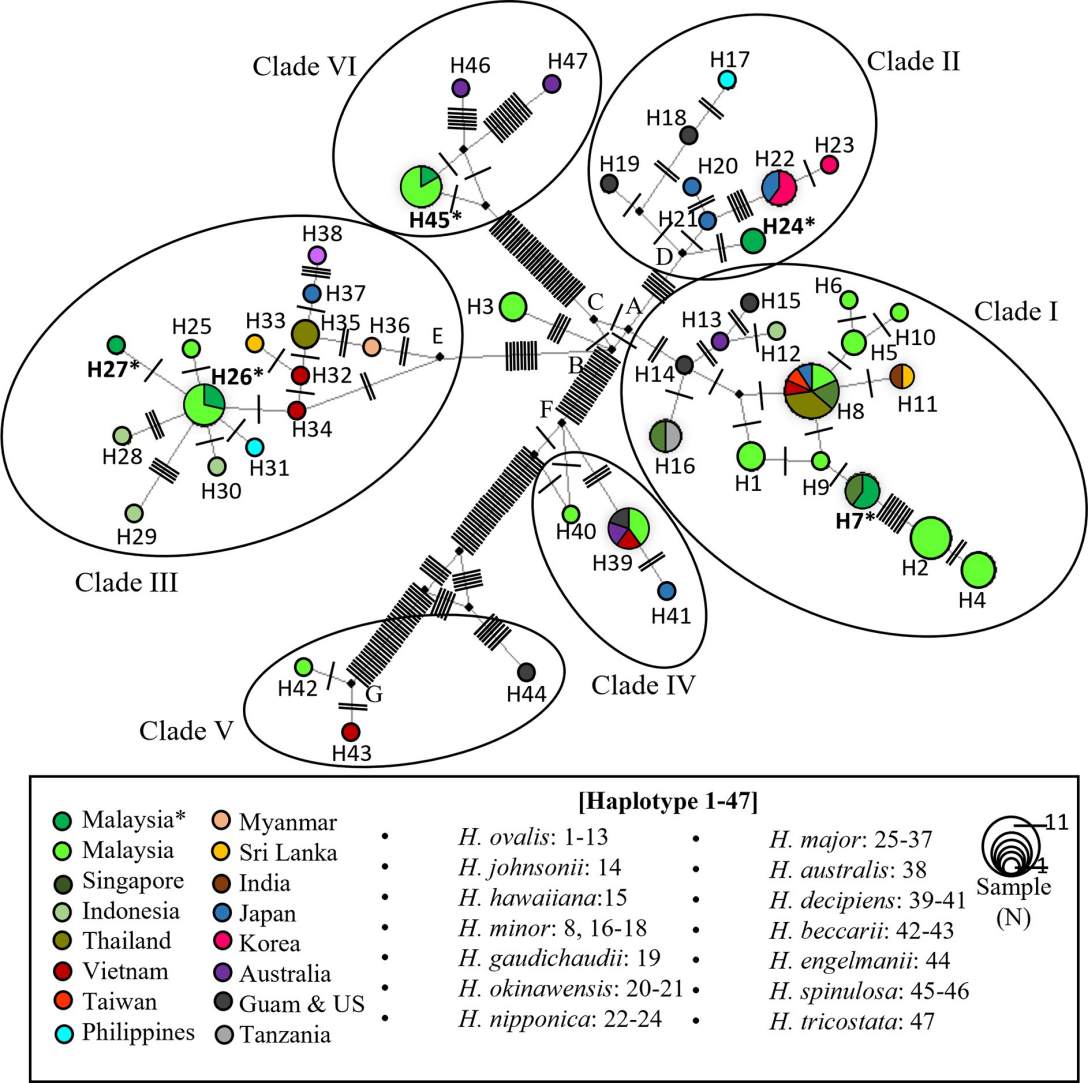
Haplotype network of *Halophila* species and related species. The circle size shows the number of samples. Mutations were designed as bars between network lines. Black dots represent root haplotype or ancestral haplotype. A-G are expected root/ancestral haplotypes for each clade. The haplotype network design was done using Network 10.2.0.0 software, and the colours were edited in Microsoft PowerPoint. (*), Samples of this study.

The sequences of all individuals were used to construct a haplotype network (Fig 9), resulting in a total of 47 haplotypes (H) from 14 species in different regions. Clade I included 16 haplotypes (N = 48), H1–H15, including *H*. *ovalis*, *H*. *minor*, *H*. *johnsonii*, and *H*. *hawaiiana*. The haplotype network of Clade II formed eight haplotypes (N = 13), H17–H24, including *H*. *nipponica*, *H*. *okinawensis*, *H*. *gaudichaudii*, and *H*. *minor*. Clade III included 14 haplotypes (N = 21), H25–H38, involving *H*. *major* and *H*. *australis*. Clades IV (*H*. *decipiens*) and V (*H*. *beccarii* and *H*. *engelmannii*) both presented three haplotypes each, H39–H41 and H42–H44, whereas Clade VI presented three haplotypes (N = 8), H45–H46 and H47, including *H*. *spinulosa* and *H*. *tricostata*.

In Clade I, the *H*. *ovalis* of this study was located at H7 along with the Singapore sample, attached from H9 (KF620346 *H*. *ovalis* Malaysia) with a single mutation/segregation site and eight mutation sites towards H2 (*H*. *ovalis* (RL) and *H*. *ovalis* (SL) from the Merambong shoal, Malaysia). *H*. *minor* from Thailand shares the same haplotype with *H*. *ovalis* on H8. *H*. *nipponica* in this study was referred to as H24, which is linked to a presumed ancestor with 24 mutation sites, connecting *H*. *okinawensis* and *H*. *nipponica* from Japan and Korea (H20–H23). Additionally, there was a genetic distance from another ancestor towards *H*. *gaudichaudii* (H19) and *H*. *minor* (H17–H18).

*H*. *major* in this study consisted of two haplotypes, H26 and H27, which differed by a single mutation site. Variant H26 was observed in the *H*. *major* samples of this study, as well as in Pulau Mabul, Pulau Salakan, and Pulau Gusungan. A sample of *H*. *major* from this study diverged as H27 from H26, indicating a potentially expanding haplotype within the population. In Clade VI, the *H*. *spinulosa* sample from this study shared H45 with Malaysian samples from Merambong shoal and Seluyong shoal in Johor. Moreover, this haplotype was found to have a shared ancestor with *H*. *spinulosa* samples from Australia, with six mutation sites differentiating them. The network haplotype constructions were a general estimation used to determine the separation between intraspecies division and mutation graphically. Specific individual comparisons of the same species across different bioregions will result in better phylogeographical determination.

A haplotype network using a universal ITS genomic region revealed closely and distantly related species. As shown in Fig 9, the haplotypes from different species formed six clusters as phylogenetic trees (Fig 8) divided by the expected root node or ancestral node. Nodes A, B, and C have one mutation/segregation from each node. The *H*. *ovalis* clade has the closest distance to the *H*. *nipponica* clade, with six mutations/segregations from node A to node G. The *H*. *major* clade has ten mutations/segregations from node B to node E. The *H*. *decipiens* clade has a greater number of mutations/segregations from node B, with 21 mutations/segregations from node B. *H*. *spinulosa* clade has 30 mutations/segregations from node C. *H*. *beccarii* clade tends to have the highest number of mutations/segregations and nodes from nodes A, B, and C.

The analysis of molecular variance (AMOVA) results shown in Table 6 revealed that 76.40% of the genetic variation was among clades, whereas 23.60% was within clades. The variance of components were 23.799 among the clades and 7.353 within the clades. The variance components were 23.799 among the clades and 7.353 within the clades. Permutation tests confirmed that both sources of variation were statistically significant (*p* < 0.05). The fixation index (F_ST_) was 0.76395, indicating substantial genetic differentiation among clades. The F_ST_ value significantly differed from the null distribution generated by permutation tests (*p* < 0.05), supporting the conclusion of high genetic differentiation.

**Table 6:** AMOVA (analysis of molecular variance) results from six clades.

Source of variation	df	Sum of squares	Variance of components	Percentage of variation	Probability
Among clades	6	1694.115	23.799	76.40	*p* < 0.05
Within clades	100	691.215	7.353	23.60	*p* < 0.05
Fixation indices (F_ST_): 0.76395					

## Discussion

### Morphometric characteristics of *Halophila* species

The morphometrics of *Halophila* species tend to be variable across different global geographic [21, 24, 46]. The morphological data for the Halophila section were compared with those for other species from other locations, as shown in S4 Table. The measurements of *H*. *major* in this study revealed longer blade lengths of up to 42.30 mm, in contrast to *H*. *major* populations from Mabul Island, East Coast of Malaysia, which typically exhibit lengths ranging between 18–22 mm [8]. Leaf lengths in other regions also varied, with Vietnam reporting values of 10–18 mm [34], Thailand reporting values of 25.9–29.4 mm [25], Indonesia reporting values of 25.04 ± 4.88 mm [29], and Ly Son Islands, Vietnam, reporting values of up to 30.66 ± 1.09 mm [26]. Compared with the samples from the present study, *H*. *major* and *H*. *ovalis* from Singapore are closer in length and width but have a slightly greater number of cross veins, with 15–21 pairs for *H*. *major* and 12–17 pairs for *H*. *ovalis* [33]. The morphological variation of *Halophila* leaves is influenced by environmental factors such as substrate, salinity, depth, and associated habitat in Malaysia [23, 24]. Accordingly, three categories were divided into big-leaved, intermediate-leaved, and small-leaved variants based on leaf size dimensions and pairs of cross veins. *H*. *ovalis* in Sarawak, found at similar depths presented small leaves on muddy substrates, intermediate leaves on sandy substrates, and big leaves on muddy–sandy substrates [23]. *H*. *ovalis* from the Merambong shoal had both small-leaved and big-leaved plants on the same substrate but were exposed to different conditions [24].

The key identification in this study lies in the ratio of the distance between the intramarginal vein and the blade margin with a half-width lamina (r:R), which allows differentiation between *H*. *major*, *H*. *ovalis*, and *H*. *nipponica* (S4 Table). *H*. *major* also resulted in a higher ratio of r:R from other regions because they have wider leaves. *H*. *major* from Southeast Asia recorded a range of 1:16.6–27.1 mm from Thailand, Malaysia, Indonesia, and Vietnam [8, 25, 29, 47], and *H*. *major* from Japan showed a similar range of 1: 20–25 mm [16]. In this study, *H*. *ovalis* was distinguished from *H*. *major* by the ratio of r:R from 1:8–14 mm, a finding that is consistent with similar observations reported in other studies [8, 16, 25]. Additionally, this study identifies the tristichous arrangement of leaflets as a distinctive characteristic of *H*. *spinulosa* (Spinulosae section), as this characteristic was initially discovered at the Tanjung Adang shoal [4].

### Genetic and haplotype diversity

Genetic and AMOVAs of multiple *Halophila* species, which are divided into six clades, revealed significant genetic differences (F_ST_: 0.76395) and varying levels of interspecies divergence, ranging from 0.032 to 0.165. *H*. *ovalis* demonstrated highly significant differences (F_ST_: 0.679) among 14 populations from different regions in the Western Pacific and Eastern Indian Oceans [8]. The use of the universal ITS marker in this study proves valuable for studying closely related species of the same genus, owing to the high degree of sequence variation and genetic differences it detects.

For example, *H*. *nipponica* exhibited a shorter distance from *H*. *ovalis* than *H*. *major* did, indicating that *H*. *ovalis* may act as an intermediate species between the two. Additionally, owing to significant morphological differences, *H*. *spinulosa* and *H*. *beccarii* presented the greatest genetic divergence within the Halophila section. Our results revealed sequence divergence, with an average of 0.044, between *H*. *ovalis* and *H*. *major* clades. *H*. *major* in Thailand was reported to have an average genetic diversity of 2.60 ± 0.40% and 4.70 ± 0.90% from *H*. *ovalis* [29]. In Vietnam, the evolutionary divergence between *H*. *ovalis* and a new record, *H*. *major*, was reported to be 0.043–0.051 [47]. In the following year, they reported that the *H*. *major* clade differed from *H*. *ovalis* by 0.033–0.038 [8]. Evolutionary divergence refers to the broader process of populations or species diverging from a common ancestor, whereas genetic divergence is a specific aspect of this process, with a focus on the accumulation of genetic differences between these divergent lineages [28, 48, 49]. Extensive genetic diversity in dominant seagrass populations can improve growth performance and stability, even when disruptions occur. Therefore, it is crucial to prioritize the preservation of high genetic diversity within seagrass populations to maintain the normal structure and functioning of the ecosystems they contribute [50]. A genetic study in *Thalassia testudinum* demonstrated high genetic diversity and strong connectivity among morphologically diverged meadows across acute physiochemical gradients, suggesting strong phenotypic plasticity as a general-purpose trait under natural selection in this species [51].

The study also revealed high haplotype diversity (Hd) for *H*. *nipponica*, *H*. *ovalis*, and *H*. *major*, ranging from 0.859 to 0.914. This result is influenced by the large sample size and wide geographical distribution, encompassing diverse environments and habitats, providing ample opportunities for genetic differentiation and the accumulation of distinct haplotypes [47]. Conversely, *H*. *decipiens*, and *H*. *spinulosa* with low sample sizes, reached 1.00 in the case of *H*. *beccarii* because of their grouping with *H*. *engelmanii*, which contains many mutations. In a study of a specific species of *H*. *major*, distinct levels of haplotype diversity (Hd) were reported, with region IV (Sri Lanka) displaying a low range of Hd (0.202), region V (Bay of Bengal and Coast of Japan) showing a mid-range of haplotype diversity (0.710–0.713), and the highest Hd values found in region II (Wallacea site: Philippines and Indonesia) and region III (Sahul Shelf sites: Australia), ranging from 0.933 to 1 [26]. In another study, *H*. *ovalis* from Southeast Asia demonstrated greater diversity than *H*. *ovalis* from the Red Sea [30]. This explains why intraspecies studies are more specific than interspecies studies, but there is a problem in yielding some clear phylogeographical separation among the regions [26].

Previous haplotype studies focused on one species, *H*. *major* [26] and *H*. *ovalis* [30], in different regions, whereas this study compared multiple species, *H*. *ovalis*, *H*. *major*, *H*. *nipponica*, and *H*. *spinulosa*, in different regions. Haplotype 8 (H8) might be the ancestral haplotype for *H*. *ovalis* and is shared by Malaysia, Singapore, Thailand, Vietnam, Taiwan, and Japan. Previous studies have shown that *H*. *ovalis* from Southeast Asia and East Asia share the same haplotype and present a high number of haplotype providers [30]. The genetic phylogeny, historical biogeography, and evolution of Hydrocharitaceae indicated that *Halophila* possibly originated in Southeast Asia 15.9–41.3 million years ago [52]. The studies of specific species revealed higher haplotype numbers than those of multiple species did, thus revealing a more detailed evolutionary history and identifying unique haplotypes. For example, *H*. *major* from 11 countries was reportedly divided into five regions with 22 haplotypes [26]. In contrast, our study identified only 14 haplotypes from 9 countries grouped into a single clade. *H*. *ovalis* from Japan were separated individually by haplotypes from Southeast Asia [30]. However, *H*. *ovalis* from East Malaysia shares the same haplotype as that from Thailand [8], which is similar to our results.

### New distribution records of *Halophila nipponica* from Malaysia

The Tropical Indo-Pacific bioregion, which extends from East Africa, Southeast Asia, and tropical Australia to the eastern Pacific, is the largest and most diverse region for tropical seagrasses [45]. Eleven *Halophila* species have been documented in Southeast Asia, including *H*. *ovalis*, *H*. *major*, *H*. *minor*, *H*. *ovata*, *H*. *gaudichaudii*, *H*. *decipiens*, *H*. *sulawesii*, *H*. *beccarii*, *H*. *spinulosa*, *H*. *tricostata* (*Halophila* sp. 2), and *Halophila* sp. 1 [7]. Although *H*. *nipponica* has not yet been identified molecularly in Southeast Asia, formal molecular identification of *H*. *minor* from the Philippines [28] has shown clustering within the *H*. *nipponica* clade [18]. Similarly, *H*. *gaudichaudii*, which was genetically identified [18] from Guam, is distributed in the Tropical Western Pacific Ocean [53]. Temperate *H*. *nipponica* from Japan grow on sandy or muddy sediment associated with *H*. *major*, *Zostera marina*, *Z*. *japonica* and *Z*. *caespitosa* [29]. These plants are able to grow not only in temperate Japan and South Korea, where the temperature is less than 20°C, but also in tropical waters, where they grow in summer [54]. Subtropical Japanese waters ranged and remained constant at 26°C in the summer months for the whole region, and there was a 3°C difference in the winter months between the southern islands [55]. The water in Malaysia exceeds 28–32°C, as *H*. *ovalis* has a wide range of tolerances to different seagrass bioregions [3, 8, 28, 30]. *H*. *nipponica* shows habitat preferences, but it has been shown to be endemic to the temperate bioregion of Japan [16].

Morphologically, *H*. *nipponica* from the Tanjung Adang Laut shoal differed from *H*. *ovalis* (Table 2). Further analysis via key identification [16, 37] revealed that this sample ranged from *H*. *nipponica* to *H*. *gaudichaudii* where the distance between the intramarginal and blade margins (r) and the ratio r:½ lamina width were overlapped, with *H*. *nipponica* from Chiba Pref. Japan (0.5–1.0 mm; 1:1.5–6.5 mm), *H*. *gaudichaudii* from Okinawa Pref. Japan (0.4–0.6 mm; 1:4.0–8.3 mm) and *H*. *gaudichaudii* from Marshall Island (0.32–0.58 mm) [16, 53]. *H*. *nipponica* from Okinawa Pref. was characterized by distinct leaf morphology across different localities, including wide leaves (elliptic type) from Ishigaki Island, intermediate leaves (linear type) from Okinawan Island, and narrower leaves (up to 1 mm wide) from Ie Island [17, 18]. In the other region, the morphological and reproductive features of *H*. *nipponica* from the coast of South Korea closely resembled those of the species in Japan [56]. *H*. *gaudichaudii* from Marshall Island [53] displayed a close morphological resemblance, featuring obovate leaves identical to those of *H*. *gaudichaudii* from Okinawa, Japan, characterized by a broader leaf type [16]. This variation in *H*. *gaudichau*dii has been treated synonymously with *H*. *nipponica* [17, 18], resembling the characteristics observed in our species. Historically, the *Halophila* section of the so-called *Halophila ovalis-minor* complex, which has elliptic and ovate leaf shapes including *H*. *ovata*, *H*. *ovalis*, *H*. *minor*, *H*. *nipponica*, *H*. *okinawensis*, and *H*. *gaudichaudii*, has been misclassified because of overlapping morphologies [16–18]. *H*. *nipponica*, *H*. *okinawensis* and *H*. *gaudichaudii* may be conspecific [17], with similar numbers of cross-veins and a rarity or absence of branched cross-veins.

Phylogenetically, *H*. *gaudichaudii* forms a distinct subclade towards *H*. *nipponica* and *H*. *okinawensis* [17], where our sample forms an outgroup from both subclades with 99% bootstrap values (Fig 8). Genetic distance analysis revealed that our sample was closest to *H*. *okinawensis* (Okinawa Pref.) with 0.8% and 1.2% (5–8 bp) differences and 1.0% (7 bp) difference from *H*. *gaudichaudii* from Guam. *H*. *gaudichaudii* exhibited a divergence of 0.8–1.7% (5–11 bp) from both *H*. *okinawensis* and *H*. *nipponica* [17]. A comparison of our sample with *H*. *nipponica* temperate Japan and South Korea revealed of 1.5% and 1.7% (10–11 bp) difference. *H*. *nipponica* from Korea is identical and has fewer than three bp differences from temperate Japanese species [19]. The distance of genetic differences revealed that subtropical species presented differences of up to 1.7% from temperate species, which is why our samples from the tropical Pacific presented the same distance. Haplotype diversity and network analysis (Fig 9; Table 5) revealed *H*. *nipponica* (including *H*. *okinawensis*, *H*. *gaudichaudii*, *H*. *minor*) from eight different haplotypes, each of which was divided by segregation or mutation into 16 total sites. Our sample exhibited a clear distinction, with one haplotype closely linked to *H*. *okinawensis* and another displaying a distant ancestral connection to *H*. *gaudichaudii*. Notably, the same haplotype was shared between *H*. *nipponica* populations from temperate Japan and Korea. The Philippines and Guam populations share the same ancestor and have been proven to occupy the same latitudinal range in Pacific water. This result indicates that seagrass bioregion have a large effect on genetic distance. However, the conspecific nature of this clade, as mentioned previously, might be due to its shared characteristics, and this clade has wide morphological barriers to its locality [17, 18]. *H*. *okinawensis* became a native species to Okinawan Pref., whereas *H*. *gaudichaudii* was distributed in subtropical Japan, Micronesia (Guam and Marshal Island), and the Philippines [16, 17, 53]. We propose that our sample represents a distinct population in the Johor region, characterized by genetic uniqueness and sequence segregation compared with temperate and subtropical waters. In light of these findings, we recommend identifying our sample as *H*. *nipponica*, which aligns with the *H*. *nipponica* clade however, it morphologically resembles *H*. *gaudichaudii*.

## Conclusions

The *Halophila* species collected from the Tanjung Adang Laut shoal were identified by morphometric and molecular genetics analysis via ITS sequences. Our finding identified *H*. *nipponica* as a new distribution record in Malaysia. Significant morphological differences (*p* < 0.05) were observed by the key of identification, with *H*. *nipponica* having the least number of paired cross-veins and greater values in the ratio of half lamina and the distance between the intramarginal vein and the blade margin, compared to *H*. *ovalis* and *H*. *major*. The ITS sequence analysis also revealed species distinctions between *H*. *ovalis*, *H*. *nipponica*, *H*. *major*, and *H*. *spinulosa* clades. Genetic distances of *H*. *nipponica* revealed high similarity to *H*. *okinawensis* and *H*. *gaudichaudii*, which were grouped in the same clades. However, *H*. *nipponica* was observed in this study with a single haplotype, thus proving genetic variation among these species. The relatively small sample size of *H*. *nipponica* suggests further research to understand its genetic diversity and distribution. A comparison of haplotype diversities revealed that *H*. *ovalis* in Clade I contains the highest number of samples but has lower haplotype diversity than Clade III of *H*. *major*. Genetic variation between *Halophila* clades is generally high and divided by their ancestral lineage and the number of mutations or segregation sites between haplotypes.

## Supporting information

S1 Table Sample collection from GenBank accession dataset for phylogenetic analyses. (DOCX)

S2 Table Estimates of evolutionary divergence between sequences. The number of base substitutions per site from between sequences are shown. Analyses were conducted using the Tamura 3-parameter model [1]. The rate variation among sites was modeled with a gamma distribution (shape parameter = 1). This analysis involved 100 nucleotide sequences. Codon positions included were 1st+2nd+3rd+Noncoding. All ambiguous positions were removed for each sequence pair (pairwise deletion option). There were a total of 651 positions in the final dataset. Evolutionary analyses were conducted in MEGA11 [2]. (*), Samples of this study; (#), Undeposited sequences from Japar Sidik Bujang (JSB).
(XLSX)

S3 Table Sequence divergences of *Halophila* species by clade. (*), Samples of this study; (#), Undeposited sequences from Japar Sidik Bujang (JSB).
(XLSX)

S4 Table The leaf morphology between *Halophila* species from Tanjung Adang Laut shoal, Johor, and different regions. (DOCX)
