# Supplementary material for: Morphological and genetic identification of Halophila species and a new distribution record of Halophila nipponica at the Tanjung Adang Laut shoal, Johor, Malaysia
Source: PLoS One. 2024 Oct 3;19(10):e0309143. doi: 10.1371/journal.pone.0309143 (PMC11449352; doi:10.1371/journal.pone.0309143)
Supplement: S1 Table — (DOCX) [file pone.0309143.s001.docx]

**Table S1. Sample collection from GenBank accession dataset for phylogenetic analyses.**

| **No.** | **Species** | **Location** | **GenBank accession** | **Source** |
| --- | --- | --- | --- | --- |
| 1. | *H. ovalis* | Johor, Malaysia | AF366420.1 | [1] |
| 2. |  | Sibangat Island, Sabah, Malaysia | KF620342.1 | [2] |
| 3. |  | Bodgaya Island, Sabah, Malaysia | KF620343.1 | [2] |
| 4. |  | Maiga Island, Sabah, Malaysia | KF620344.1 | [2] |
| 5. |  | Johor, Malaysia | KF620346.1 | [2] |
| 6. |  | Singapore | OQ597093.1 | [3] |
| 7. |  | Singapore | OQ597120.1 | [3] |
| 8. |  | Singapore | OQ597146.1 | [3] |
| 9. |  | Flores Island, Kajouwulu, Indonesia | AB436930.1 | [4] |
| 10. |  | Trang, Thailand | KF620351.1 | [2] |
| 11. |  | Koh Tan, Suratthani, Thailand | KP408241.1 | [5] |
| 12. |  | Trang, Thailand | KX668192.1 | [6] |
| 13. |  | Van Phong Bay, Vietnam | KC175912.1 | [2] |
| 14. |  | Gulf of Mannar, Sri Lanka | MT347907.1 | [7] |
| 15. |  | Chilika, India | KM609942.1 | [8] |
| 16. |  | Daguoye, Taiwan | OM478532.1 | [9] |
| 17. |  | Okinawa, Japan | AF366421.1 | [1] |
| 18. |  | Moreton Bay, Australia | AF366418.1 | [1] |
| 19. | *H. major* | Mabul Island, Sabah, Malaysia | KF620340.1 | [2] |
| 20. |  | Gusungan Island, Sabah, Malaysia | KF620341.1 | [2] |
| 21. |  | Sindhu Beach, Bali, Indonesia | AB436928.1 | [4] |
| 22. |  | Tual, Indonesia | MT028355.1 | [10] |
| 23. |  | Lombok, Indonesia | MT028356.1 | [10] |
| 24. |  | Trang, Thailand | AB436927.1 | [4] |
| 25. |  | Trang, Thailand | KF620348.1 | [2] |
| 26. |  | Ao Nang, Krabi, Thailand | KP408265.1 | [5] |
| 27. |  | Panglao, Philippines | MT586875.1 | [11] |
| 28. |  | Nha Trang Bay, Vietnam | KC175910.1 | [12] |
| 29. |  | Vietnam | MW084347.1 | [13] |
| 30. |  | Gyeiktaw, Myanmar | KF620352.1 | [2] |
| 31. |  | Sri Lanka | MT347897.1 | [7] |
| 32. |  | Kagoshima, Japan | AB436929.1 | [4] |
| 33. | *H. minor* | Singapore | OQ597097.1 | [3] |
| 34. |  | Singapore | OQ831660.1 | [3] |
| 35. |  | Singapore | OQ831661.1 | [3] |
| 36. |  | Trang, Thailand | KX668191.1 | [6] |
| 37. |  | Philippines | AF366406.1 | [1] |
| 38. |  | Guam | AF366405.1 | [1] |
| 39. |  | Tanzania | ON684467.1 | [14] |
| 40. |  | Tanzania | ON684474.1 | [14] |
| 41. | *H. nipponica* | Simane, Japan | AB436935.1 | [4] |
| 42. |  | Okinawa, Japan  (*H. okinawensis*)* | AB436936.1 | [4] |
| 43. |  | Okinawa, Japan  (*H. okinawensis*)* | AB436937.1 | [4] |
| 44. |  | Hiroshima, Japan | AB523410.1 | [4] |
| 45. |  | South Korea | HQ687164.1 | Unpublished |
| 46. |  | Goheung County, South Korea | KX668185.1 | [6] |
| 47. |  | Gyeongsangnam-do, South Korea | KX668188.1 | [6] |
| 48. |  | Jeju, South Korea | KX668190.1 | [6] |
| 49. |  | Guam  (*H. gaudichaudii*)* | AB436924.1 | [4] |
| 50. | *H. decipiens* | Malaysia | AF366412.1 | [1] |
| 51. |  | Johor, Malaysia | MN200776.1 | [15] |
| 52. |  | Nha Trang Bay, Vietnam | KC175913.1 | [12] |
| 53. |  | Okinawa, Japan | AB243983.1 | [16] |
| 54. |  | Queenland, Australia | AF366411.1 | [1] |
| 55. |  | Midway Atoll, USA | AF395673.1 | [17] |
| 56. | *H. australis* | Albany, Australia | AF366414.1 | [1] |
| 57. | *H. johnsonii* | Florida, USA | AF366425.1 | [1] |
| 58. | *H. hawaiiana* | Hawaii, USA | AF366426.1 | [1] |
| 59. | *H. beccarii* | Vietnam | AF366441.1 | [1] |
| 60. | *H. engelmannii* | Florida, USA | AF366404.1 | [1] |
| 61. | *H. spinulosa* | Malaysia | AF366440.1 | [1] |
| 62. |  | Australia | AF366439.1 | [1] |
| 63. | *H. tricostata* | Australia | AF366438.1 | [1] |

* Original name

References

1. Waycott M, Freshwater DW, York RA, Calladine A, Kenworthy WJ. Evolutionary trends in the seagrass genus *Halophila* (Thouars): insights from molecular phylogeny. Bull Mar Sci. 2002;71(3): 1299-1308. Available from: <https://www.ingentaconnect.com/contentone/umrsmas/bullmar/2002/00000071/00000003/art00016>
2. Nguyen VX, Detcharoen M, Tuntiprapas P, Soe-Htun U, Japar Sidik B, Muta Harah Z, et al. Genetic species identification and population structure of *Halophila* (Hydrocharitaceae) from the Western Pacific to the Eastern Indian Ocean. BMC Evol Biol. 2014;14: 92. doi: [10.1186/1471-2148-14-92](https://doi.org/10.1186/1471-2148-14-92). PMID: [24886000](https://pubmed.ncbi.nlm.nih.gov/24886000/).
3. Kwan V, Shantti P, Lum EYY, Ow YX, Huang D. Diversity and phylogeny of seagrasses in Singapore. Aquat Bot. 2023;187: 1-10. doi: [10.1016/j.aquabot.2023.103648](https://doi.org/10.1016/j.aquabot.2023.103648).
4. Uchimura M, Faye EJ, Shimada S, Inoue T, Nakamura Y. A reassessment of *Halophila* species (Hydrocharitaceae) diversity with special reference to Japanese representatives. Bot Mar. 2008;51(4): 258-268. doi: [10.1515/BOT.2008.036](https://doi.org/10.1515/BOT.2008.036).
5. Tuntiprapas P, Shimada S, Pongparadon S, Prathep A, Saensouk P, Theerakulpisut P, et al. Is *Halophila major* (Zoll.) Miquel a big *H. ovalis* (R. Brown) J. D. Hooker? An evaluation based on age, morphology, and ITS sequence. Sci Asia. 2015;41(2): 79-86. doi:[10.2306/scienceasia1513-1874.2015.41.079](https://www.scienceasia.org/content/viewabstract.php?ms=5893).
6. Kim YK, Kim SH, Yi JM, Kang CK, Short F, Lee KS. Genetic identification and evolutionary trends of the seagrass *Halophila nipponica* in temperate coastal waters of Korea. PLoS One. 2017;12(5): e0177772. doi: [10.1371/journal.pone.0177772](https://doi.org/10.1371/journal.pone.0177772). PMID: [28505209](https://pubmed.ncbi.nlm.nih.gov/28505209/).
7. Liu SYV, Kumara TP, Hsu CH. Genetic identification and hybridization in the seagrass genus *Halophila* (Hydrocharitaceae) in Sri Lankan waters. PeerJ. 2020;8: e10027. doi: [10.7717/peerj.10027](https://doi.org/10.7717/peerj.10027). PMID: [33062439](https://pubmed.ncbi.nlm.nih.gov/33062439/).
8. Nguyen XV, Höfler S, Glasenapp Y, Thangaradjou T, Lucas C, Papenbrock J. New insights into DNA barcoding of seagrasses. Syst Biodivers. 2015;13(5): 496-508. doi: [10.1080/14772000.2015.1046408](https://doi.org/10.1080/14772000.2015.1046408).
9. Liu SYV, Hsu CH, Li CY. Temporal instability in genetic structure of a marine monocot (*Halophila ovalis*). Estuar Coast Shelf Sci. 2022;276: 1-8. doi: [10.1016/j.ecss.2022.108034](https://doi.org/10.1016/j.ecss.2022.108034).
10. Kurniawan F, Imran Z, Darus RF, Anggraeni F, Damar A, Sunuddin A, et al. Rediscovering *Halophila major* (Zollinger) Miquel (1855) in Indonesia. Aquat Bot. 2020;161: 1-4. doi: [10.1016/j.aquabot.2019.103171](https://doi.org/10.1016/j.aquabot.2019.103171).
11. Kolátková V, Čepička I, Hoffman R, Vohník M. Marinomyxa Gen. Nov. Accommodates Gall-Forming Parasites of the Tropical to Subtropical Seagrass Genus *Halophila* and Constitutes a Novel Deep-Branching Lineage Within Phytomyxea (Rhizaria: Endomyxa). Microb Ecol. 2021;81(3): 673-686. doi: [10.1007/s00248-020-01615-5](https://doi.org/10.1007/s00248-020-01615-5). PMID: [33021677](https://pubmed.ncbi.nlm.nih.gov/33021677/).
12. Nguyen VX, Holzmeyer L, Papenbrock J. New record of the seagrass species *Halophila major* (Zoll.) Miquel in Vietnam: evidence from leaf morphology and ITS analysis. Bot Mar. 2013;56(4): 313-321. doi: [10.1515/bot-2012-0188](https://doi.org/10.1515/bot-2012-0188).
13. Nguyen XV, Nguyen-Nhat NT, Nguyen XT, Dao VH, M Liao L, Papenbrock J. Analysis of rDNA reveals a high genetic diversity of *Halophila major* in the Wallacea region. PLoS One. 2021;16(10): e0258956. doi: [10.1371/journal.pone.0258956](https://doi.org/10.1371/journal.pone.0258956). PMID: [34679102](https://pubmed.ncbi.nlm.nih.gov/34679102/).
14. Lusana JL, Robert Lugendo B. Delineating seagrass species in the genera *Halodule* and *Halophila* from Tanzanian coastal waters using ITS and rbcL DNA barcoding. Nord J Bot. 2023;3: e03823. doi: [10.1111/njb.03823](https://doi.org/10.1111/njb.03823).
15. Rozaimi M, Arina N, Zainee NFA, Raynusha C, Hidayah N. An uncommon intertidal record of *Halophila decipiens* Ostenfeld in the vicinity of mangroves. Aquat Bot. 2020;160: 1-5. doi: [10.1016/j.aquabot.2019.103168](https://doi.org/10.1016/j.aquabot.2019.103168).
16. Uchimura M, Faye EJ, Shimada S, Ogura G, Inoue T, Nakamura Y. A taxonomic study of the seagrass genus *Halophila* (Hydrocharitaceae) from Japan: description of a new species *Halophila japonica* *sp. nov*. and characterization of *H. ovalis* using morphological and molecular data. Bull Natn Sci Mus. 2006;32(3): 129-150. Available from: <https://www.kahaku.go.jp/research/publication/botany/download/32_3/BNSM_B320304.pdf>
17. McDermid KJ, Gregoritza MC, Freshwater DW. A new record of a second seagrass species from the Hawaiian archipelago: *Halophila decipiens* Ostenfeld. Aquat Bot. 2002;74(3): 257-262. doi: [10.1016/S0304-3770(02)00081-5](https://doi.org/10.1016/S0304-3770(02)00081-5).
