# Supplementary material for: Morphological and genetic identification of Halophila species and a new distribution record of Halophila nipponica at the Tanjung Adang Laut shoal, Johor, Malaysia
Source: PLoS One. 2024 Oct 3;19(10):e0309143. doi: 10.1371/journal.pone.0309143 (PMC11449352; doi:10.1371/journal.pone.0309143)
Supplement: S4 Table — (DOCX) [file pone.0309143.s004.docx]

**Table S4. The leaf morphology between *Halophila* species from Tanjung Adang Laut shoal, Johor, and different regions.**

| **Species**  **Location** | **Vegetative structure dimension** | | | | | | | | | **References** |
| --- | --- | --- | --- | --- | --- | --- | --- | --- | --- | --- |
|  | **BL (mm)** | **BW (mm)** | **ratio BL: BW (mm)** | **R (mm)** | **r (mm)** | **ratio r:R (mm)** | **NCV** | **ACV** | **DBCV (mm)** |  |
| *H. nipponica*  Tanjung Adang Laut shoal, Johor  Population 1 | 9.11±0.59  (8.21–10.35) | 6.33±0.27  (5.95–7.00) | 1.44±0.06  (1.34–1.58:1) | 3.29±0.18  (3.06–3.57) | 0.61±0.05  (0.52–0.69) | 5.46±0.41  (1:4.76–6.13) | 5.67±1.18  (4–7) | 66.20°±8.65  (50.25°–82.86°) | 1.55±0.62  (0.59–2.50) | This study |
| *H. nipponica*  Tanjung Adang Laut shoal, Johor  Population 2 | 8.93±0.46  (8.32–9.92) | 6.07±0.56  (5.18–7.26) | 1.48±0.11  (1.29–1.64:1) | 3.17±0.31  (2.61–3.75) | 0.59±0.06  (0.52–0.70) | 5.36±0.39  (1:4.89–6.02) | 5.60±0.91  (4–7) | 65.29°±6.48  (52.37°–73.51°) | 1.41±0.66  (0.43–2.29) | This study |
| *H. nipponica*  Chiba Pref., Japan | (12-)18–25(-30) | (3-)5–9(-13) | (2-)3–4(-6): 1 | n/d | 0.5–1 | 1:1.5–6.5 | (6-)7–9(-10) | 30°–45° | 1.4–2 | [1] |
| *H. nipponica*  Misaki, Japan | (12-)15–23(-28) | (4.5-)7–9(-13) | (2.5)3–4: 1 | n/d | n/d | n/d | n/d | n/d | n/d | [1] |
| *H. nipponica*  Noto, Japan | (17-)20–25(30) | (3-)4–6(-8) | 3–5(-6):1 | n/d | n/d | n/d | n/d | n/d | n/d | [1] |
| *H. nipponica*  Kyushu, Japan | (14-)18 | (6-)8–10 | 2.0–2.2(-2.5) :1 | n/d | n/d | n/d | n/d | n/d | n/d | [1] |
| *H. nipponica* | Up to 3 | Up to 1 | n/d | n/d | 0.1–0.2 | n/d | 2–4 | n/d | n/d | [2] |
| *H. nipponica*  South Korea | 25.2 ± 0.3 (19.8–32.0) | 10.6 ± 0.2 (8.4–15.9) | n/d | n/d | n/d | n/d | 11.0 ± 0.1  (9–13) | n/d | n/d | [3] |
| *H. okinawensis*  Okinawa, Japan | 12–16 | 1.5-4 | (2.5-)3.5–4(-7) :1 | n/d | 0.25–0.3 | 1:2–9.5 | (5-)6–7 | n/d | 2 | [1] |
| *H. gaudichaudi*  Okinawa, Japan | (8-)10–15(-17) | (3.5-)4–6(-8) | (1.6-)2–2.4(-2.6): 1 | n/d | 0.4–0.6 | 1:4.0–8.3 | (3-)4–8 | 30°–45° | 1.7 | [1] |
| *H. gaudichaudi*  Marshall Island | 7–14 | 4–6 | n/d | n/d | 0.32–0.58 | n/d | Less than 8 | n/d | n/d | [4] |
| *H. minor*  Japan | 10 | 4–5 | 1.8–2.2:1 | n/d | 0.15–0.4 | 1:11.5–16 | 7–12 | n/d | 0.65–0.85 | [1] |
| *H. minor*  Pulau Perhentian, Terengganu, Malaysia | 11.00–19.70 | 5.90–12.10 | 1.37–2.08:1 | n/d | 0.18–0.36 | n/d | 7–11 | n/d | 0.46–2.55 | [5] |
| *H. minor*  Pulau Redang, Terengganu, Malaysia | 13.62 ± 0.18  (11.14–17.98) | 8.25 ± 0.12  (5.73–10.52) | 1.67 ± 0.02:1  (1.27–2.35:1) | n/d | 0.18 ±0.01  (0.14–0.29) | n/d | 9.00 ± 0.16  7–11 | n/d | 0.70 ± 0.02  (0.57–1.00) | [6] |
| *H. minor*  Singapore | 7.05 ± 0.71  5.93–7.93 | 3.50 ± 0.59  2.53–4.46 | n/d | n/d | 0.1–0.3 | n/d | 7-12 | n/d | n/d | [7] |
| *H. ovalis*  Tanjung Adang Laut shoal, Johor  Population 1 | 11.25±1.07  (9.86–13.13) | 6.43±0.52  (5.70–7.67) | 1.75±0.11  (1.57–1.90:1) | 3.28±0.28  (2.88–3.91) | 0.33±0.04  (0.25–0.42) | 10.12±1.35  (1:8.56–13.56) | 10.62±1.85  (7–14) | 65.74±7.64  (50.55°–77.13°) | 0.99±0.52  (0.13–1.81) | This study |
| *H. ovalis*  Tanjung Adang Laut shoal, Johor  Population 2 | 11.94±1.25  (9.55–14.38) | 7.08±0.69  (5.31–8.20) | 1.69±0.13  (1.45–1.87:1) | 3.65±0.35  (2.84–4.24) | 0.37±0.04  (0.32–0.46) | 9.85±1.12  (1:8.29–11.46) | 9.47±1.25  (8–11) | 66.51±7.93  (52.39°–86.89°) | 1.08±0.58  (0.24–1.98) | This study |
| *H. ovalis*  Tanjung Adang Laut shoal, Johor  Population 3 | 13.75±1.20  (11.77–15.32) | 8.05±0.97  (6.62–10.15) | 1.72±0.19  (1.39–1.96:1) | 4.12±0.56  (3.12–5.42) | 0.37±0.06  (0.28–0.49) | 11.28±1.86  (1:8.00–14.16) | 10.47±1.25  (8–12) | 63.00±5.92  (53.04°–70.9°) | 1.23±0.57  (0.31–1.98) | This study |
| *H. ovalis*  Pantai Bangat, Lawas, Sarawak, Malaysia | 20.57±4.07  (7.91–10.88) | 10.44±1.18  (8.38-12.86) | n/d | n/d | n/d | n/d | 14.45±2.35  (10–19) | n/d | n/d | [8] |
| *H. ovalis*  Pantai Bangat, Lawas, Sarawak, Malaysia | 16.19±2.1  (9.03–10.34) | 8.72±0.73  (7.55–10.13) | n/d | n/d | n/d | n/d | 11.6±1.81  (8–15) | n/d | n/d | [8] |
| *H. ovalis*  Pantai Bangat, Lawas, Sarawak Malaysia | 12.31±1.15  (4.30–10.38) | 6.72±0.76  (5.63–8.89) | n/d | n/d | n/d | n/d | 10.65±1.87  (7–15) | n/d | n/d | [8] |
| *H. ovalis*  Punang, Sarawak, Malaysia | 19.7±0.18 16.29–23.10 | 8.98±0.09 7.09–10.57 | n/d | n/d | n/d | n/d | 14.48±0.20 12–18 | n/d | n/d | [9] |
| *H. ovalis*  Merchang, Terengganu, Malaysia | 17.24±0.18 (10.2–24.70) | 7.82±10.09 (4.62–13.35) | n/d | n/d | n/d | n/d | 12.64±10.07  (8–18) | n/d | n/d | [9] |
| *H. ovalis*  Merambong, Johor, Malaysia | 11.36±10.09 (6.68–15.89) | 6.54±10.05 (3.56–9.65) | n/d | n/d | n/d | n/d | 9.78±0.06 (7–15) | n/d | n/d | [9] |
| *H. ovalis*  Pulau Gaya, Sabah, Malaysia | 8.191±0.12 (2.08–14.03) | 5.47±0.07 (2.21–9.31) | n/d | n/d | n/d | n/d | 9.66±0.07 (6–16) | n/d | n/d | [9] |
| *H. ovalis*  Teluk Kemang, Negeri Sembilan, Malaysia | 13.1±0.17 (5.11–21.01) | 7.14±0.07 (3.10–11.89) | n/d | n/d | n/d | n/d | 10.91±0.0(6 8–15) | n/d | n/d | [9] |
| *H. ovalis*  Merchang, Terengganu, Malaysia | 9.36±2.13  (4.56–14.02) | 4.33±1.24  (1.55–7.40) | n/d | n/d | 0.26±0.07  (0.10–0.47) | n/d | 8.94±1.76  (3–14) | n/d | 1.15±5.24  (0.30–81) | [10] |
| *H. ovalis*  Singapore | 12.50 ± 5.05  (8.09–24.54) | 5.56 ± 1.98  (3.97–9.77) | n/d | n/d | 0.2–0.3 | n/d | 12-17 | n/d | n/d | [7] |
| *H. ovalis*  Andaman Sea, Thailand | 10.0–15.0 | 4.6–7.6 | n/d | n/d | 0.2–0.6 | 1:6.8–12.1 | 9–14 | 43.4°–79.4° | 0.3–1.1 | [11] |
| *H. ovalis*  Vietnam | 9–12 | 3.7–7.0 | n/d | n/d | 0.3 | 1:9–17 | 8–16 | 45°–80° | n/d | [12] |
| *H. ovalis*  Japan | 10–18 | 4–10 | 1.3–2.0:1 | n/d | 0.25–0.4 | 1:10–16 | 12–16 | n/d | n/d | [1] |
| *H. major*  Tanjung Adang Laut shoal, Johor  Population 1 | 30.83±2.58  (26.81–37.95) | 17.67±1.49  (15.10–21.65) | 1.75±0.09  (1.62–1.93:1) | 9.14±0.75  (7.99–11.30) | 0.45±0.05  (0.40–0.59) | 20.53±1.99  (1:17.98–24.08) | 14.73±0.88  (13–16) | 56.86±4.15  (50.22°–64.79°) | 2.53±1.38  (0.51–4.38) | This study |
| *H. major*  Tanjung Adang Laut shoal, Johor  Population 2 | 38.36±2.22  (35.11–42.30) | 19.40±1.86  (15.80–22.03) | 2.00±0.23  (1.69–2.36:1) | 10.08±0.82  (8.59–11.22) | 0.47±0.09  (0.30–0.58) | 22.30±5.54  (1:17.33–36.80) | 14.69±1.37  (13–18) | 58.51±5.37  (48.95°–66.37°) | 3.25±1.92  (0.77–5.73) | This study |
| *H. major*  Tanjung Adang Laut shoal, Johor  Population 3 | 33.91±2.50  (28.82–37.25) | 19.14±1.46  (15.21–20.86) | 1.78±0.13  (1.50–2.03:1) | 9.98±0.72  (8.31–11.09) | 0.40±0.06  (0.30–0.55) | 25.59±4.39  (1:18.75–36.97) | 15.53±1.18  (14–18) | 65.04±5.96  (53.59°–72.05°) | 2.72±1.50  (0.46–4.66) | This study |
| *H. major*  Mabul Island, Sabah, Malaysia | 18 – 22 | 12 – 15 | n/d | n/d | 0.25 – 0.3 | 1:21 – 22 | 18 – 20 | n/d | n/d | [13] |
| *H. major*  Gusungan Island, Sabah, Malaysia | 18 – 22 | 12 – 15 | n/d | n/d | 0.25 – 0.3 | 1:21 – 22 | 18 – 20 | n/d | n/d | [13] |
| *H. major*  Myanmar | 22 | 13 | n/d | n/d | 0.3 | 1:20 | 20 | n/d | n/d | [13] |
| *H. major*  Singapore | 28.15 ± 6.88  (17.2–36.2) | 11.29 ± 4.99  (7.7–16.4) | n/d | n/d | 0.2–3.7 | n/d | 15–21 | n/d | n/d | [7] |
| *H. major*  Ly Son Island, Vietnam | 30.66±1.09 | 10.12±0.36 | n/d | n/d | n/d | n/d | 16–17 | 45–60 | 1.54±0.32 | [14] |
| *H. major*  Phu Quoc Island, Vietnam | 18.10±0.26 | 8.32±0.30 | n/d | n/d | n/d | n/d | 14–17 | 45–60 | 0.91±0.10 | [14] |
| *H. major*  Con Dao Island, Vietnam | 11.46±0.11 | 6.84±0.17 | n/d | n/d | n/d | n/d | 19–22 | 75–80 | 0.80±0.21 | [14] |
| *H. major*  Thailand | 23.9–29.4 | 10.8–12.6 | n/d | n/d | 0.1-0.6 | 1:16.6–27.1 | 14-18 | n/d | n/d | [11] |
| *H. major*  Tayondo, Indonesia | 25.04 ± 4.88 | 13.48 ± 3.25 | n/d | n/d | 0.35 ± 0.08 | 1:19.02 ± 1.38 | 19 ± 2 | 73.18 ± 2.08 | 0.86 ± 0.21 | [15] |
| *H. major*  Tual, Indonesia | 22.84 ± 0.91 | 12.26 ± 0.19 | n/d | n/d | 0.33 ± 0.01 | 1:18.49 ± 0.40 | 19 ± 2 | 64.92 ± 2.71 | 0.82 ± 0.10 | [15] |
| *H. major*  Lombok, Indonesia | 20.24 ± 4.26 | 9.68 ± 1.09 | n/d | n/d | 0.26 ± 0.02 | 1:20.82 ± 2.68 | 18 ± 2 | 82.26 ± 6.08 | 0.91 ± 0.21 | [15] |
| *H. major*  Toli-toli, Indonesia | 20.89 ± 2.88 | 12.59 ± 2.26 | n/d | n/d | 0.32 ± 0.08 | 1:19.92 ± 2.30 | 15 ± 2 | 70.83 ± 5.47 | 0.98 ± 0.18 | [15] |

BL, Blade length; BW, Blade width; R, half lamina width; r, distance between intramarginal and blade margin; NCV, number of paired cross veins; ACV, angle of the cross vein; DBCV, distance between cross veins. n/d, no data.

References

1. Kuo J, Kanamoto Z, Iizumi H, Mukai H. Seagrasses of the genus *Halophila* thouars (Hydrocharitaceae) from Japan. Acta Phytotaxon Geobot. 2006;57(2): 129-154. doi: [10.18942/apg.KJ00004622858](https://doi.org/10.18942/apg.KJ00004622858).
2. Shimada S, Watanabe M, Ichihara K, Uchimura M. Morphological variations of seagrass species, *Halophila nipponica* (Hydrocharitaceae, Alismatales). Coast Mar Sci. 2012;35: 85-90. doi: [10.15083/00040639](https://doi.org/10.15083/00040639).
3. Kim JB, Park JI, Jung CS, Lee PY, Lee KS. Distributional range extension of the seagrass *Halophila nipponica* into coastal waters off the Korean peninsula. Aquat Bot. 2009;90(3): 269-272. doi: [10.1016/j.aquabot.2008.10.007](https://doi.org/10.1016/j.aquabot.2008.10.007).
4. Tsuda RT, Sukhraj N. Reassessment of seagrass species in the Marshall Islands. Micronesica. 2016;4: 1-10. Available from: <https://micronesica.org/sites/default/files/tsudasukhraj_proof_vfinalv2.pdf>
5. Muta Harah Z, Japar Sidik B. Occurrence and distribution of seagrasses in waters of Perhentian Island archipelago, Malaysia. J Fish Aquat Sci. 2013;8(3): 441-451. doi: [10.3923/jfas.2013.441.451](https://doi.org/10.3923/jfas.2013.441.451).
6. Muta Harah Z, Japar Sidik B, Abdul RFR. Occurrence and morphological description of seagrasses from Pulau Redang, Terengganu, Malaysia. J Teknol. 2003;38: 29–39. doi: [10.11113/jt.v38.491](https://doi.org/10.11113/jt.v38.491).
7. Kwan V, Shantti P, Lum EYY, Ow YX, Huang D. Diversity and phylogeny of seagrasses in Singapore. Aquat Bot. 2023;187: 1-10. doi: [10.1016/j.aquabot.2023.103648](https://doi.org/10.1016/j.aquabot.2023.103648).
8. Japar Sidik B, Muta Harah Z, Mohd FI, Khairul, AM. Growth performance of Malaysian's spoongrass, *Halophila ovalis* (R. Br.) Hooker *f*. under different substrates, salinity and light regime. Coast Mar Sci. 2010;34(1): 103-107. doi: [10.15083/00040678](https://doi.org/10.15083/00040678).
9. Annaletchumy L, Japar Sidik B, Muta Harah Z, Arshad A. Morphology of *Halophila ovalis* (R. Br.) Hook. *f.* from Peninsular and East Malaysia. Pertanika J Trop Agric Sci. 2005;28(1): 1-11. Available from: <http://www.pertanika.upm.edu.my/pjtas/browse/regular-issue?article=JTAS-0296-2005>
10. Japar Sidik B, Muta Harah Z, Arshad A. Morphological characteristics, shoot density and biomass variability of *Halophila* spp. in a coastal lagoon of east coast of Malaysia. Coast Mar Sci. 2010;34(1): 108-12. doi: [10.15083/00040679](https://doi.org/10.15083/00040679).
11. Tuntiprapas P, Shimada S, Pongparadon S, Prathep A, Saensouk P, Theerakulpisut P, et al. Is *Halophila major* (Zoll.) Miquel a big *H. ovalis* (R. Brown) J. D. Hooker? An evaluation based on age, morphology, and ITS sequence. Sci Asia. 2015;41(2): 79-86. doi:[10.2306/scienceasia1513-1874.2015.41.079](https://www.scienceasia.org/content/viewabstract.php?ms=5893).
12. Nguyen VX, Japar Sidik B, Papenbrock J. Variability of leaf morphology and marker genes of members of the *Halophila* complex collected in Viet Nam. Aquat Bot. 2013;110: 6-15. doi: [10.1016/j.aquabot.2013.04.003](https://doi.org/10.1016/j.aquabot.2013.04.003).
13. Nguyen VX, Detcharoen M, Tuntiprapas P, Soe-Htun U, Japar Sidik B, Muta Harah Z, et al. Genetic species identification and population structure of *Halophila* (Hydrocharitaceae) from the Western Pacific to the Eastern Indian Ocean. BMC Evol Biol. 2014;14: 92. doi: [10.1186/1471-2148-14-92](https://doi.org/10.1186/1471-2148-14-92). PMID: [24886000](https://pubmed.ncbi.nlm.nih.gov/24886000/).
14. Nguyen XV, Nguyen-Nhat NT, Nguyen XT, Dao VH, M Liao L, Papenbrock J. Analysis of rDNA reveals a high genetic diversity of *Halophila major* in the Wallacea region. PLoS One. 2021;16(10): e0258956. doi: [10.1371/journal.pone.0258956](https://doi.org/10.1371/journal.pone.0258956). PMID: [34679102](https://pubmed.ncbi.nlm.nih.gov/34679102/).
15. Kurniawan F, Imran Z, Darus RF, Anggraeni F, Damar A, Sunuddin A, et al. Rediscovering Halophila major (Zollinger) Miquel (1855) in Indonesia. Aquat Bot. 2020;161: 1-4. doi: 10.1016/j.aquabot.2019.103171.
